# Epigenetic Modifiers Induce Bioactive Phenolic Metabolites in the Marine-Derived Fungus *Penicillium brevicompactum*

**DOI:** 10.3390/md16080253

**Published:** 2018-07-30

**Authors:** Seham S. El-Hawary, Ahmed M. Sayed, Rabab Mohammed, Hossam M. Hassan, Mohamed A. Zaki, Mostafa E. Rateb, Tarek A. Mohammed, Elham Amin, Usama Ramadan Abdelmohsen

**Affiliations:** 1 Pharmacognosy Department, Faculty of Pharmacy, Cairo University, Cairo 11787, Egypt; seham.elhawary@yahoo.com; 2 Pharmacognosy Department, Faculty of Pharmacy, Beni-Suef University, Beni-Suef 62514, Egypt; Ahmedpharma8530@gmail.com (A.M.S.); rmwork06@yahoo.com (R.M.); abuh20050@yahoo.com (H.M.H.); m_abuzaki@yahoo.com (M.A.Z.); Mostafa.Rateb@uws.ac.uk (M.E.R.); elham.amin@nub.edu.eg (E.A.); 3 Pharmacognosy Department, Faculty of Pharmacy, Nahda University, Beni-Suef 62513, Egypt; 4 Marine Biodiscovery Centre, University of Aberdeen, Aberdeen, Scotland AB24 3UE, UK; 5 School of Computing, Engineering and Physical Sciences, University of the West of Scotland, Paisley PA1 2BE, UK; 6 Marine Invertebrates, National Institute of Oceanography and Fisheries, Red Sea Branch, Hurghada 84511, Egypt; tare_mote@yahoo.com; 7 Department of Pharmacognosy, Faculty of Pharmacy, Minia University, Minia 61519, Egypt

**Keywords:** HDACs inhibitors, *Penicillium brevicompactum*, phenolic metabolites, nicotinamide, sodium butyrate, antiproliferative, pharmacophore

## Abstract

Fungi usually contain gene clusters that are silent or cryptic under normal laboratory culture conditions. These cryptic genes could be expressed for a wide variety of bioactive compounds. One of the recent approaches to induce production of such cryptic fungal metabolites is to use histone deacetylases (HDACs) inhibitors. In the present study, the cultures of the marine-derived fungus *Penicillium brevicompactum* treated with nicotinamide and sodium butyrate were found to produce a lot of phenolic compounds. Nicotinamide treatment resulted in the isolation and identification of nine compounds **1**–**9**. Sodium butyrate also enhanced the productivity of anthranilic acid (**10**) and ergosterol peroxide (**11**). The antioxidant as well as the antiproliferative activities of each metabolite were determined. Syringic acid (**4**), sinapic acid (**5**), and acetosyringone (**6**) exhibited potent in vitro free radical scavenging, (IC_50_ 20 to 30 µg/mL) and antiproliferative activities (IC_50_ 1.14 to 1.71 µM) against HepG2 cancer cell line. Furthermore, a pharmacophore model of the active compounds was generated to build up a structure-activity relationship.

## 1. Introduction

Numerous diseases, including cancer, male infertility, heart diseases, Alzheimer’s disease, and ageing are linked to uncontrolled oxidative stress [1,2]. Antioxidants such as phenolic compounds inhibit the formation of free radicals and maintain the normal redox regulation of the human body [3]. Such facts encouraged the search for antioxidant and anticancer compounds from natural sources [4]. In fungi, secondary metabolites associated genes are expressed in response to biotic and abiotic environmental factors [5]. Under standard laboratory conditions, the gene clusters that regulate secondary metabolism in fungi are often transcriptionally silenced. To activate such gene clusters in fungi, several strategies have been developed [6,7]. The OSMAC (One Strain of Many Compounds) strategy has been developed in the early 2000s as a simple and effective way to increase the diversity of fungal secondary metabolites [8]. In this strategy, silent genes were activated by modification of culture conditions, including media composition, UV irradiation, shaking and incubation temperature [9,10]. One of the prevalent epigenetic mechanisms influencing gene transcription is histone modification. Histone proteins are responsible for maintaining chromatin structure either in an accessible or inaccessible state to different transcriptional activators for their binding to gene promoters [11]. Acetylation of histones by histone acetyltransferases (HATs) makes the chromatin more open and accessible for transcription factors which in turn increase the expression of secondary metabolites producing genes. Conversely, deacetylation which is performed by histone deacetylases (HDACs) induces chromatin condensation and gene inactivation [12,13]. Three classes of HDACs (I, II and III) have been identified in fungi [14]. Recently, the ability of small molecules such as sodium butyrate and nicotinamide to inhibit the catalytic activity of HDACs and to induce the expression of secondary metabolites has been shown in fungi [15,16,17,18,19,20,21]. In the present study, we investigated the effect of two different histone deacetylase (HDAC) inhibitors (nicotinamide and sodium butyrate) on the production of bioactive secondary metabolites by the sponge-derived fungus *Penicillium brevicompactum.*

## 2. Results and Discussion

### 2.1. Molecular Identification of Isolated Marine Fungus

The amplified internal transcribed spacer (ITS) and beta-tubulin region of the isolated fungal strain CALYF1 (GenBank accession number for ITS sequence: MH300130, and for beta-tubuline sequence: MH560351) was sequenced and compared with the ITS and beta-tubulin sequences of microorganisms represented in the National Centre for Biotechnology Information (NCBI) database gene bank using Basic Local Alignment Search Tool (BLAST) search. ITS and beta-tubuline sequences analysis showed 99% identity with *Penicillium brevicompactum* strain AI-F-DRBC-1 (Genbank accession No. MH101383.1) and 99% identity with *Penicillium brevicompactum* strain CBS (Genbank accession No. AY674437.1), respectively. Therefore, strain CALYF1 isolated here was identified as *Penicillium brevicompactum.*

### 2.2. Total Phenolic Content

For primary screening, the total phenolic content (TPC) was determined in all fungal extracts derived from different culture conditions. The TPC was expressed in gallic acid equivalents (GAE) per mg of extract. Table 1 showed that the fungal extracts derived after treatment with HDAC inhibitors (nicotinamide and sodium butyrate) contained the highest TPC among extracts of all cultures.

### 2.3. Isolation and Characterization of Induced Metabolites

**Figure 1:**
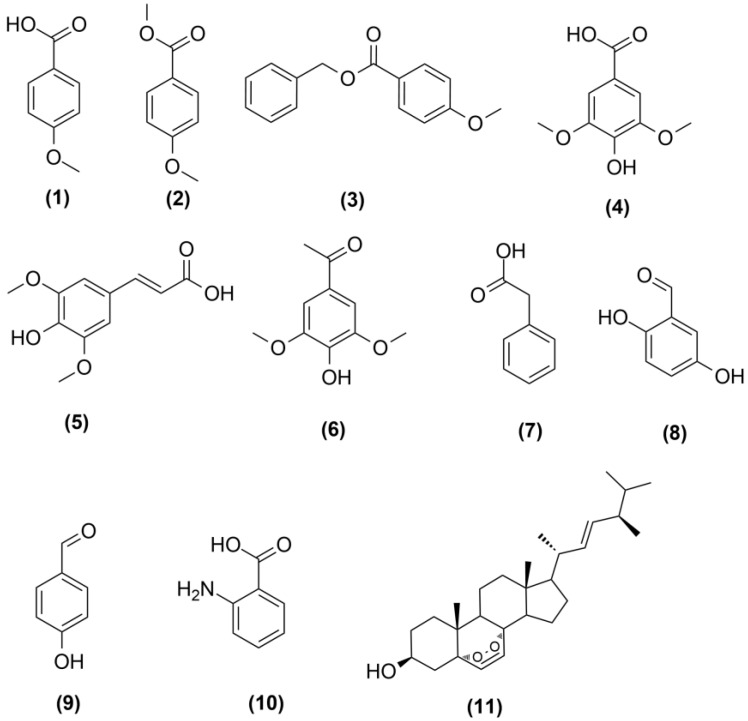
Induced fungal metabolites after treatment with HDAC inhibitor.

Bioactive fungal extract derived from nicotinamide treatment were further purified by Sephadex LH20 followed by reversed-phase C_18_ column chromatography to isolate nine compounds. These compounds were identified as *p*-anisic acid (**1**) [22], *p*-anisic acid methyl ester (**2**) [23], benzyl anisate (**3**) [24], syringic acid (**4**) [25], sinapic acid (**5**) [26], acetosyringone (**6**) [25], phenyl acetic acid (**7**) [27], gentisaldehyde (**8**) [28] and *p*-hydroxy benzaldehyde (**9**) [26] (Figure 1). Chromatographic purification of fungal extract obtained from sodium butyrate treatment led to isolation of two compounds that were identified as anthranilic acid (**10**) [25] and ergosterol peroxide (**11**) [29] (Figure 1). All the isolated compounds were identified by comparison of their ^1^H, ^13^C NMR and mass spectrometric (MS) spectral data with those reported in the literature (Figures S1–S38). Additionally, the presence of compounds **1**, **2**, **3**, **7**, **8** and **9** in the fungal extract derived from sodium butyrate treatment was confirmed by high-performance liquid chromatography (HPLC) analysis. None of the aforementioned compounds could be detected in the untreated cultures. We can conclude from the previous discussion that each HDAC inhibitor interacts with different histone deacetylases and with varying degrees.

### 2.4. Free Radical Scavenging Activity

The DPPH radical scavenging activities of isolated compounds (**1**–**11**) were measured (Table 2). Syringic acid (**4**), acetosyringone (**6**) and sinapic acid (**5**) showed strong DPPH radical scavenging activities (IC_50_ 20 ± 0.09, 25 ± 0.05 and 30 ± 0.08 μg/mL, respectively) comparable to the positive control ascorbic acid and gallic acid, (IC_50_, 10 and 15 ± 0.05 μg/mL). Gentisaldehyde (**8**) and *p*-hydroxy benzaldehyde (**9**) showed a moderate DPPH radical scavenging activity (IC_50_ 75 ± 0.02 and 95 ± 0.08 μg/mL, respectively). The other compounds did not show scavenging activity up to 200 μg/mL. The above-mentioned data suggest that the presence of *p*-hydroxy (–OH) group is essential for the radical scavenging activity, which is further improved by the presence of methoxy groups (–OCH_3_).

### 2.5. Antiproliferative Activity against Human Liver Cancer (HepG2)

Gallic acid is well known for its antioxidant activity. Moreover, it has a selective antiproliferative activity against many cancer types [30,31,32,33]. So far, no previous in vitro studies discussing the antiproliferative activity of the isolated compounds against human liver cancer were found. Accordingly, the cytotoxic activity of different concentrations of the isolated compounds, together with gallic acid as internal structure-related standard, against HepG2 cancer cell line was assessed. Results (Table 3) revealed, that compounds (**4**–**6**) together with gallic acid could effectively decrease cell viability in a dose dependent manner as effective as (or closer to) Doxorubicin (positive control) in HepG2 cancer cell line. These results are in accordance with the results of the radical scavenging activity that indicated highest activity of the same compounds (Table 2). A previous study reported that the cytotoxicity exhibited by gallic acid is not a prevailing feature in all phenolic compounds. Moreover, this activity is attributed to the presence of three adjacent hydroxy groups in gallic acid molecule and not due to the presence of the carboxyl group [33]. Based on the previous data, these results suggest that the structural similarity between these compounds and gallic acid along with their strong antioxidant activity are the most important determinants for their antiproliferative activity.

### 2.6. 3D Alignment and Pharmacophore Generation

**Figure 2:**
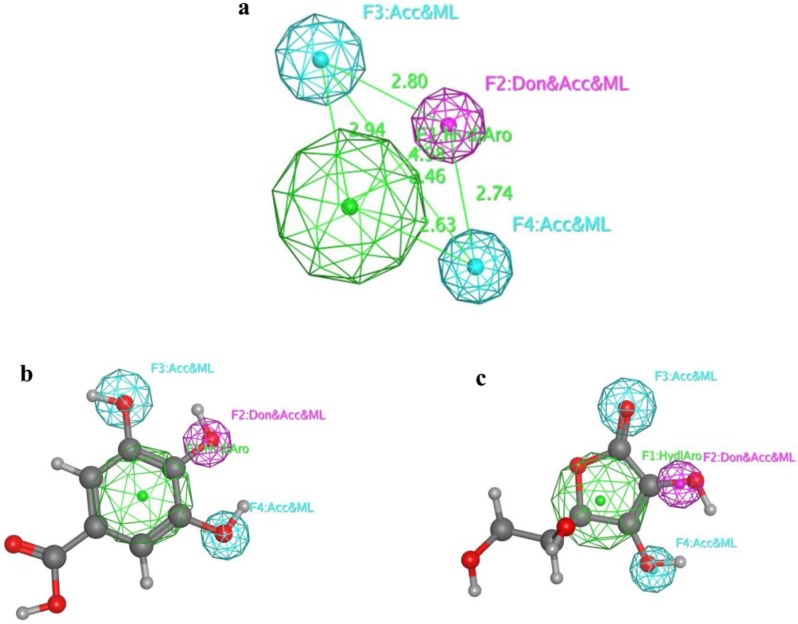
The best MOE pharmacophore model (Hypo 1). (**a**) Chemical features present in Hypo 1; (**b**) Mapping of gallic acid on Hypo 1; (**c**) Mapping of ascorbic acid on Hypo 1.

To date, the exact target and mechanism of cytotoxicity of gallic acid and its related compounds are poorly established [30,31,33]. Therefore, a ligand-based pharmacophore modeling approach was chosen to produce a predictive model for antioxidant phenolic compounds. In this method, a model is based on the common chemical features of the active compounds. The automatic construction and visualization of the 3D pharmacophore models from the structural data of the most active compounds **4**–**6** (training set) was created by MOE 2018 software and the results of the six top-scored 3D hypothetical pharmacophores generation are shown (Supplementary Table S1) with their statistical parameters. Figure 2 shows the highest ranked pharmacophore model (Hypo 1) including the chemical features with spatial relationship and geometric parameters. In Hypo 1, the pharmacophore is characterized by two features of hydrogen bond acceptor (HBA), one hydrogen bond donor or acceptor and one aromatic hydrophobic function. After mapping the remaining compounds along with gallic acid (external standard) and ascorbic acid (internal standard) as a test set onto the generated pharmacophore, Hypo 1 recognized only the active compounds gallic acid and ascorbic acid, as they completely fitted with the essential features of the pharmacophore (Figure 2). In conclusion, we suggested that the newly generated pharmacophore model may have the predictive capacity for further phenolic antioxidant and antiproliferative hits.

### 2.7. HPLC Profiles of Induced Phenolics

**Figure 3:**
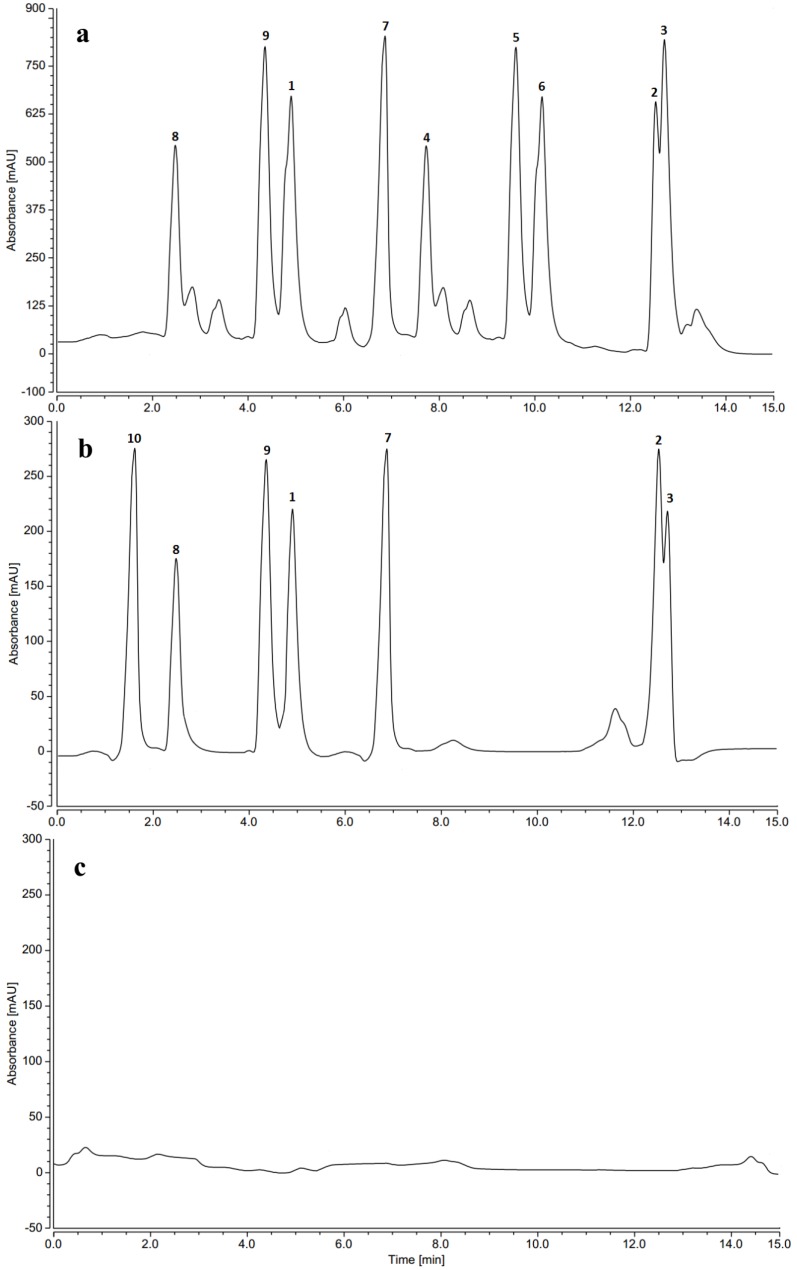
HPLC profiles of fungal extracts obtained from malt extract culture after, (**a**) nicotinamide treatment; (**b**) sodium butyrate treatment; (**c**) without HDAC inhibitor treatments. HPLC isocratic elution was performed with 60% aqueous acetonitrile containing 1% formic acid as a mobile phase at a flow rate of 1 mL/min and UV detection at 254 nm. Peaks **1**–**10** represent the isolated metabolites. Compound **11** was detected at 210 nm, so it did not appear in these chromatograms.

Phenolic profiles of all fungal extracts were determined by HPLC (Figure 3) using the isolated fungal metabolites as reference standards for qualitative and quantitative analysis of each extract. The concentration of each compound in different fungal extracts is presented in Table 4. Generally, HPLC profiles (Figure 3) showed that extracts of HDAC inhibitor treated cultures had higher metabolite diversity than the untreated extracts. Additionally, nicotinamide treatment resulted in higher phenolics concentrations than sodium butyrate treatment (Table 4). None of the phenolic compounds could be detected in the untreated fungal extracts (Supplementary Figure S39). Furthermore, syringic acid (**4**), sinapic acid (**5**) and acetosyringone (**6**) were not traced in the fungal extract of sodium butyrate treatment indicating that nicotinamide induced additional metabolic pathway for production of these metabolites. Treatment with varying concentrations of nicotinamide and sodium butyrate affected only on the concentration of the phenolic metabolites produced. HPLC analysis illustrated that fungal cultures treated with 100 µM nicotinamide produced the maximum quantities of phenolic metabolites (Table 5). Like nicotinamide, the highest phenolic concentrations were recorded in the fungal culture subjected to 0.01 M sodium butyrate.

## 3. Experimental

### 3.1. Isolation of Marine Fungal Strain

The marine sponge sample *Callyspongia siphonella* was collected from Shaab Saad area at 13 km northern Hurghada along the Red Sea Coast (GPS coordinates N 27°15′48″, E 33°49′3″) at depth of 5–7 m in November 2015. A voucher specimen (NIOF204/2015) was reserved at the National Institute of Oceanography and Fisheries, Red Sea Branch, Invertebrates Department. Sponges were transferred to plastic bags containing seawater and transported to the laboratory for further processing. Sponge specimens were rinsed in sterile seawater, cut into small pieces, and then thoroughly homogenized in a sterile mortar with 10 volumes of sterile seawater. The supernatant was diluted in ten-fold series (10^−1^, 10^−2^, 10^−3^) and subsequently plated out on malt agar plates (Lobachemie^®^, Mumbai, Maharashtra, India) supplemented with ampicillin (0.5 mg mL^−1^) to suppress bacterial growth. All the plates were incubated at 25 ± 2 °C and were regularly monitored for any mycelia growth [34,35]. Pure fungal isolates were obtained upon repeated subculturing and were kept at 4 °C.

### 3.2. Fungal Strain Identification

Taxonomic identification of the fungal strain was achieved by genomic DNA extraction, amplification and sequencing of the fungal ITS and beta-tubulin regions according to Samson et al., 2004 protocol [36]. Obtained Sequences were submitted to GenBank, NCBI with accession number for ITS sequence: MH300130, and for beta-tubulin sequence: MH560351. Alignment with published sequences in GenBank showed that the fungal strain had 99% identity with *Penicillium brevicompactum* strain AI-F-DRBC-1 (Genbank accession No. MH101383.1) and 99% identity with *Penicillium brevicompactum* strain CBS (Genbank accession No. AY674437.1), respectively. The isolated fungal strain was deposited (code: Pen-011) in the Microbiology Department, School of Pharmacy, Nahda University, Egypt.

### 3.3. Set up Growing Condition and Crude Extract Production

To investigate the effects of different culture media on secondary metabolites production, *P. brevicompactum* was first activated in malt extract agar for 3 days. Then, a single colony from malt agar was inoculated in 150 mL malt extract broth (malt extract 15 g/L) for 3 days. Finally, 30 mL of this culture was inoculated in 5 L Erlenmeyer flasks containing 1.5 L of five different culture media. The five different types of media were made as follows: malt extract broth (15 g malt extract and deionized water to 1 L), malt extract with artificial sea water (15 g malt extract, NaCl 23.5 g, Na_2_SO_4_ 4 g, NaHCO_3_ 0.2 g, KBr 0.1 g, and deionized water to 1 L), Sabouraud Dextrose broth (40 g dextrose, 10 g peptone and deionized water to 1 L), Czapek Dox broth (30 g sucrose, NaNO_3_ 2 g, 1 g K_2_HPO_4_, Mg_2_SO_4_ 0.5 g, 0.5 NaCl, 0.01 FeSO_4_, and deionized water to 1 L), and rice medium (100 mL of deionized water were added to 100 g commercially available shelled rice and kept overnight prior to autoclaving). After four weeks of static fermentation in dark at 25 ± 2 °C, secondary metabolites were extracted from the five different culture media with ethyl acetate. To study the effect of HDAC inhibitor on the secondary metabolites productivity of this fungus, a single colony from malt agar was inoculated in 150 mL malt extract broth (malt extract 15 g/L) for 3 days. 30 mL of this culture were inoculated in 500 mL Erlenmeyer flasks containing 150 mL malt extract broth treated with 10, 50, 100 and 500 µM of nicotinamide (Lobachemie^®^), and another 30 mL were inoculated into 500 mL Erlenmeyer flasks containing 150 mL malt extract supplemented with 0.005, 0.01, 0.015 and 0.02 M of sodium butyrate (Alfa Aesar^®^, Ward Hill, Massachusetts, USA). The flasks were incubated under static conditions in the dark at 25 ± 2 °C. After four weeks, the culture broths of nicotinamide and sodium butyrate treatment were extracted by ethyl acetate. The extracts were concentrated under reduced pressure and then subjected to HPLC analysis. For preparative upscaling, *P. brevicompactum* was cultivated using 5 L Erlenmeyer flasks containing 1.5 L malt extract broth in the presence of nicotinamide 100 µM or sodium butyrate 0.01 M, and after four weeks the whole broth from both treatments were extracted twice with ethyl acetate and concentrated under reduced pressure to obtain the dry extracts.

### 3.4. Chemical Analysis and Instrumentation

Ultraviolet spectra were acquired on an ultraviolet/visible (UV-vis) spectrometer (Shimadzu UV 1800 spectro, Kyoto, Japan). HPLC analysis was performed by Thermofisher dionex ultimate 3000 with UV- vis detector and Xterra (Waters, Milford, Massachusetts, USA) C_18_ RP analytical HPLC column (5 µm, 4.6 × 250 mm). Detailed 1D and 2D NMR spectra were recorded on Bruker Avance III 400 MHz (Bruker AG, Fallanden, Zurich, Switzerland) with BBFO Smart Probe and Bruker 400 AEON Nitrogen-Free Magnet. Data were analyzed using Topspin 3.1 Software. Each sample was dissolved in suitable deuterated solvent. Chemical shifts were recorded and expressed in ppm related to the tetramethylsilane (TMS) signal at 0.00 ppm as internal reference. The ESI-MS data were obtained on an Agilent series 1100 SL (Agilent CO, Santa Clara, California, USA). All solvent used for preparing extracts were of technical grade (ADWIC; El-Nasr Pharmaceutical Chemicals Co., El Khanka, Kaliobeya, Egypt); and reagents used for preparing samples were of analytical grade (E-Merck, Darmstadt, Germany). Silica gel (60–120 mesh, 50 g, Sigma Aldrich^®^, St. Louis, Missouri) was used for chromatographic isolation and purification.

### 3.5. Determination of Antioxidant Activity

The free radical scavenging capacity of the isolated compounds was determined using DPPH (2,2-diphenyl-1-picrylhydrazyl) method [37,38]. Scavenging activity of the DPPH free radical was calculated using the following formula:

Inhibition % = 100(*Ac* − *As*)/*Ac*

where the percentage inhibition value was calculated from the absorbance of the control, *Ac*, and of the sample, *As*. A curve of the % inhibition against the concentration of the samples was prepared to obtain the IC_50_. The controls contained all the reaction reagents except the extract or positive control substance. The values are presented as the means of triplicate analyses. Nicotinamide and sodium butyrate did not show any free radical scavenging activity, indicating that the residual quantity of them after fermentation did not contribute to the antioxidant potential of the extracts.

### 3.6. Determination of TPC

The TPC assay was conducted based on the Folin-Ciocalteu’s phenol reagent method [39,40]. The TPC was calculated using a calibration curve for gallic acid, and was expressed as the gallic acid equivalent per mg of dry fungal extract. The experiment was performed in triplicate.

### 3.7. Isolation and Identification of Induced Metabolites

Ethyl acetate extract (1.1 g) obtained from nicotinamide treated fungal culture was fractionated on a Sephadex LH20 (32–64 µm, 100 × 25 mm, Fluka^®^) column eluting with MeOH/H_2_O (90:10%), to yield 6 fractions. Fraction Nr. 3 (380 mg) was subjected to C_18_ reversed-phase column chromatography (0.04–0.063 mm, 50 × 10 mm, Merck^®^) with an isocratic elution using acetonitrile/water (20:80) to give nine purified compounds **1** (60 mg), **2** (32 mg), **3** (35 mg), **4** (30 mg), **5** (15 mg), **6** (9 mg), 7 (28 mg), **8** (11 mg) and **9** (17 mg). Fungal ethyl acetate (600 mg) extract obtained from sodium butyrate treatment was also fractionated on a Sephadex LH20 column eluting with MeOH/H_2_O (90:10), to yield crude anthranilic acid **10** (11 mg) and ergosterol peroxide **11** (17 mg). Ergosterol peroxide was further purified by silica gel column chromatography (60–120 mesh, 50 g, Fluka^®^) using hexane/EtOAc (80:20). Structure elucidation of the isolated compounds was performed by comparison of their MS and NMR spectral data with those reported in the literature.

### 3.8. 3-(4,5-Dimethylthiazol-2-yl)-2,5-Diphenyltetrazolium Bromide (MTT) Cytotoxicity Assay

Human hepatocellular carcinoma (HepG2) cell line was supplied from the American Type Cell Culture Collection (ATCC, Manassas, VA, USA) and cultured in Roswell Park Memorial Institute medium 1640 (RPMI1640) with 10% *v*/*v* inactivated fetal calf serum, 1% glutamine, 1% nonessential amino acids, 10 mg mL^−1^ streptomycin, and100 U mL^−1^ penicillin. Cells were kept in humidified atmosphere at 37 °C. The full medium was changed two times per week. At 80% confluence, cells were trypsinized and seeded in 24-well plates containing cell density of 1.5 × 10^4^. Cell viability was analyzed by the reduction of 2-(4,5-dimethylthiazol-2-yl)-2,5-diphenyltetrazolium bromide (MTT) into formazan. Cells were exposed to different compounds at the desired concentrations, (0.01, 0.1, 1, 10, and 100 µM) or 1% dimethyl sulfoxide (DMSO) for 24 h. MTT was mixed with the culture medium at a final concentration of 0.5 mg/mL. After incubation for 2 h at 37 °C, the media were carefully removed and replaced with 100 μL DMSO for each well. The absorbance (OD) values were determined by spectrophotometry at 490 nm. Each experiment was performed 3 independent times. Results were expressed as mean ± standard deviation.

### 3.9. Pharmacophore Model Generation

The biologically most active phenolic compounds (**4**–**6**) were converted to 3D structures and their energy minimized with the Merck Molecular Force Field (MMFF) using an energy threshold value of 15 kcal/mol above the global energy minimum until a local energy minimum was reached. The pharmacophore models were created for the aligned molecules using common feature pharmacophore generation in MOE 2018. Pharmacophore sites of a ligand are represented in the 3D space by a set of points. During the pharmacophore hypothesis generation, pharmacophoric features such as HBA, hydrogen bond donor (HBD), hydrophobic feature (Hyd), and aromatic region (Ar) were used to create reliable pharmacophore sites for the energy calculated ligands for our experimental results. The different pharmacophore hypothesis produced was further examined by using a scoring function so that it produced the best alignment of the ligands. Finally, the best pharmacophore model was used for mapping all the compounds.

### 3.10. High-Performance Liquid Chromatography (HPLC) Analysis

All isolated compounds were analyzed and quantified in fungal extracts derived from both OSMAC and HDACs inhibitor experiments. For this purpose, HPLC method was applied with slight modification [41]. Stock standard solutions containing each isolated compound (1 mg·mL^−1^) was prepared in 60% aqueous acetonitrile. The stock solutions were appropriately diluted to obtain a series of working solutions which were stored at −4 °C and were brought to room temperature before use. 2 mg from each fungal extract was dissolved in 60% aqueous acetonitrile in a 10 mL volumetric flask. Then 1 mL was filtered through a 0.22 µm membrane filter before introduced to HPLC system. Then, isocratic elution was performed with 60% aqueous acetonitrile containing 1% formic acid as a mobile phase over 15 min at a flow rate of 1 mL/min and UV detection at different wavelengths (210, 254, 270 and 300 nm). The calibration curve obtained from plotting peak areas vs. different concentrations of the standards was used to estimate the concentration of induced fungal phenolic compounds in each extract. Each standard concentration and fungal extract was injected in triplicate and the relative standard deviation (RSD %) was taken as a measure of precision, repeatability and stability.

### 3.11. Statistical Analysis

The HDACs inhibitor experiments were performed using three replicates per treatment. All measurements were performed in triplicate, and data were reported as the mean ± standard error (SE).

## 4. Conclusions

Treatment of the marine-derived fungus *P. brevicompactum* with HDAC inhibitors (nicotinamide and sodium butyrate) resulted in an induction of phenolic metabolites production. Processing of nicotinamide treatment led to isolation of nine (**1**–**9**) phenolic compounds; however, sodium butyrate treatment led to the isolation of only compounds (**10**, **11**). Compounds **4**–**6** exhibited potent in vitro free radical scavenging and antiproliferative activities. A pharmacophore model was carried out using compounds **4**–**6**, validation of this model was shown through testing the antioxidant activity of gallic acid and comparing it with the prediction power of the model. Testing of the compounds **1**–**11**, as well as gallic acid for their antiproliferative activities against HepG2 cancer cell line showed that compounds **4**–**6**, which exhibited the highest antioxidant activity in DPPH assay, were also active as antiproliferative agents. Consequently, these findings suggest a similar mode of action of these compounds. From this study, it would be concluded that nicotinamide treatment is a potential tool for the induction of cryptic genes for bioactive secondary metabolites production in fungi. Additionally, each HDACs inhibitor affected the fungal biosynthetic machinery in different ways.

## Figures and Tables

**Table 1:** Total phenolic content of fungal extracts.

Tested Extract	Total Phenolic Content ± S.D. (µg GAE/mg) ^a^
Nicotinamide Ext.	**93.4 ± 0.12**
Butyrate Ext.	**19.6 ± 0.05**
Malt Ext.	9.5 ± 0.05
Malt + salt Ext.	11.5 ± 0.05
Sabouraud Ext.	7.5 ± 0.02
Czapek Dox Ext.	8.7 ± 0.02
Rice Ext.	15.1 ± 0.05

^a^ Calculated as mg of gallic acid equivalent (GAE) per mg of dry extract, values are presented as the mean of triplicates ± standard deviation (S.D.).

**Table 2:** DPPH radical scavenging activities of isolated fungal metabolites.

Fungal Metabolite	IC_50_ ± S.D. (µg/mL)
*p*-Anisic acid (**1**)	>200
*p*-Anisic acid methyl ester (**2**)	>200
Benzyl anisate (**3**)	>200
Syringic acid (**4**)	**20 ± 0.09**
Sinapic acid (**5**)	**30 ± 0.05**
Acetosyringone (**6**)	**25 ± 0.08**
Phenyl acetic acid (**7**)	>200
Gentisaldehyde (**8**)	75 ± 0.02
*p*-Hydroxy benzaldehyde (**9**)	95 ± 0.08
Anthranilic acid (**10**)	>200
Ergosterol peroxide (**11**)	>200
**Gallic acid**	**15 ± 0.05**
Ascorbic acid	10 ± 0.05

**Table 3:** Antiproliferative activity of isolated fungal metabolites against HepG2 cancer cells.

% Viability of Human HepG2 Cell Line	Concentration µM					IC_50_
	0.01	0. 1	1	10	100	
*p*-Anisic acid (**1**)	107.8 ± 1.15	95.3 ± 1.14	86.2 ± 6.39	59.15 ± 2.8	34.84 ± 5.4	25.1
*p*-Anisic acid methyl ester (**2**)	121.3 ± 7.12	100.8 ± 6.33	133.3 ± 3.85	214.6 ± 6.73	201.8 ± 4.71	>100
Benzyl anisate (**3**)	141.15 ± 5.49	100.41 ± 5.45	121.81 ± 4.25	79.83 ± 2.37	58.23 ± 0.74	>100
Syringic acid (**4**)	**108.02 ± 7.61**	**80.59 ± 2.29**	**54.45 ± 3.72**	**18.94 ± 3.07**	**1.99 ± 2.69**	**1.22**
Sinapic acid (**5**)	**108.43 ± 8.62**	**88.62 ± 4.94**	**60.66 ± 2.88**	**20.27 ± 3.39**	**3.29 ± 1.088**	**1.71**
Acetosyringone (**6**)	**102.51 ± 2.57**	**79.21 ± 6.19**	**53.55 ± 2.08**	**19.92 ± 1.34**	**2.88 ± 0.20**	**1.14**
Phenyl acetic acid (**7**)	120.57 ± 1.91	88.27 ± 3.75	108.02 + 6.26	110.08 ± 0.82	93.20 ± 9.88	>100
Gentisaldehyde (**8**)	139.71 ± 3.14	109.46 ± 2.26	118.93 ± 2.96	149.17 ± 6.1	118.93 ± 4.51	>100
*p*-Hydroxy benzaldehyde (**9**)	96.70 ± 6.98	99.17 ± 4.83	73.45 ± 6.79	52.46 ± 9.14	60.08 ± 2.3	>100
Anthranilic acid (**10**)	86.00 ± 6.26	51.44 ± 2.37	61.11 ± 2.33	91.15 ± 4.73	94.23 ± 3.311	>100
Ergosterol peroxide (**11**)	98.71 ± 3.19	88.72 ± 2.52	54.45 ± 1.34	30.65 ± 2.26	3.70 ± 0.35	1.84
Gallic acid	**106.79 ± 4.54**	**86.60 ± 1.96**	**59.60 ± 4.2**	**17.20 ± 1.28**	**2.08 ± 0.54**	**1.53**
Doxorubicin	91.52 ± 1.40	80.82 ± 0.46	41.92 ± 1.77	33.45 ± 0.77	8.02 + 0.708	0.7

Data of the table represent mean ± S.D. of the mean.

**Table 4:** Phenolic metabolites concentrations in fungal extracts using high-performance liquid chromatography (HPLC) analysis.

Fungal Metabolite	Concentration ± S.D. (mg/L)						
	Nicotinamide Ext.	Butyrate Ext.	Malt Ext.	Malt + Salt Ext.	Sabouraud Ext.	Czapek Dox Ext.	Rice Ext.
*p*-Anisic acid (**1**)	25.6 ± 1.6	5.6 ± 0.5	-	-	-	-	-
*p*-Anisic acid methyl ester (**2**)	12.1 ± 0.7	2.3 ± 0.9	-	-	-	-	-
Benzyl anisate (**3**)	15.7 ± 1.3	1.8 ± 1.4	-	-	-	-	-
Syringic acid (**4**)	19.9 ± 0.2	-	-	-	-	-	-
Sinapic acid (**5**)	9.8 ± 0.8	-	-	-	-	-	-
Acetosyringone (**6**)	8.1 ± 1.1	-	-	-	-	-	-
Phenyl acetic acid (**7**)	14.4 ± 0.3	1.7 ± 0.7	-	-	-	-	-
Gentisaldehyde (**8**)	5.3 ± 0.6	2.7 ± 1.4	-	-	-	-	-
*p*-Hydroxy benzaldehyde (**9**)	7.1 ± 1.2	2.1 ± 0.8	-	-	-	-	-
Anthranilic acid (**10**)	-	9.8 ± 0.1	-	-	0.6 ± 0.2	0.2 ± 0.4	3.2 ± 0.2
Ergosterol peroxide (**11**)	-	11.2 ± 0.3	-	-	0.9 ± 0.6	-	2.7 ± 0.8

Nicotinamide Ext., fungal extract derived from nicotinamide treatment; Butyrate Ext., extract derived from sodium butyrate treatment; Malt Ext., fungal extract derived from culture on malt extract; Malt + salt Ext., fungal extract derived from culture on malt extract with artificial sea water; Sabouraud Ext., fungal extract derived from culture on Sabouraud Dextrose; Czapek Dox Ext.; fungal extract derived from culture on Czapek Dox media; Rice Ext., fungal extract derived from culture on rice.

**Table 5:** Effect of nicotinamide and sodium butyrate concentrations on fungal productivity of phenolic metabolites.

Fungal Metabolite	Nicotinamide Concentrations (µM)				Sodium Butyrate Concentrations (M)			
	10	50	100	500	0.005	0.01	0.015	0.02
*p*-Anisic acid (**1**)	4.4 ± 0.4	16.8 ± 0.8	25.6 ± 1.6	21.9 ± 0.9	3.2 ± 0.2	5.6 ± 0.5	4.5 ± 0.8	4.2 ± 0.3
*p*-Anisic acid methyl ester (**2**)	2.3 ± 0.2	4.8 ± 1.3	12.1 ± 0.7	13.1 ± 0.6	0.3 ± 0.1	2.3 ± 0.9	2.1 ± 1.2	1.9 ± 0.8
Benzyl anisate (**3**)	1.8 ± 0.6	6.9 ± 0.9	15.7 ± 1.3	10.9 ± 0.9	0.2 ± 0.1	1.8 ± 0.4	2.2 ± 0.4	1.9 ± 0.9
Syringic acid (**4**)	3.1 ± 1.1	13.8 ± 0.5	19.9 ± 0.2	18.3 ± 0.3	-	-	-	
Sinapic acid (**5**)	0.9 ± 1.3	3.1 ± 0.4	9.8 ± 0.8	8.5 ± 0.6	-	-	-	
Acetosyringone (**6**)	0.2 ± 0.2	2.7 ± 0.6	8.1 ± 1.1	5.5 ± 0.2	-	-	-	
Phenyl acetic acid (**7**)	2.5 ± 0.7	3.7 ± 0.8	14.4 ± 0.3	12.4 ± 0.7	0.3 ± 1.3	1.7 ± 0.7	1.4 ± 0.5	1.3 ± 0.6
Gentisaldehyde (**8**)	0.7 ± 0.6	1.1 ± 0.1	5.3 ± 0.6	3.8 ± 0.8	1.6 ± 0.8	2.7 ± 1.4	2.4 ± 0.3	3.1 ± 0.2
*p*-Hydroxy benzaldehyde (**9**)	3.2 ± 0.6	1.4 ± 0.6	7.1 ± 1.2	6.6 ± 0.7	0.2 ± 0.2	2.1 ± 0.8	1.1 ± 0.6	1.2 ± 0.5
Anthranilic acid (**10**)	-	-	-	-	1.7 ± 0.7	9.8 ± 0.1	7.7 ± 0.3	8.1 ± 0.4
Ergosterol peroxide (**11**)	-	-	-	-	7.9 ± 0.6	11.2 ± 0.3	16.5 ± 0.7	15.2 ± 1.4

Concentration of each fungal metabolite is presented as the mean of triplicates (mg/L) ± S.D.

## Supplementary Materials

The following are available online at http://www.mdpi.com/1660-3397/16/8/253/s1, Figures S1–S39: ESI-MS, 1D and 2D NMR spectra of isolated compounds.
