# Supplementary material for: Epigenetic Modifiers Induce Bioactive Phenolic Metabolites in the Marine-Derived Fungus Penicillium brevicompactum
Source: Mar Drugs. 2018 Jul 30;16(8):253. doi: 10.3390/md16080253 (PMC6117726; doi:10.3390/md16080253)
Supplement: Supplementary file 1 [file marinedrugs-16-00253-s001.pdf]

SUPPLEMENTARY MATERIAL

Epigenetic modifiers induce bioactive phenolic metabolites in the marine-derived fungus *Penicillium brevicompactum*.

Seham S. El-Hawary[1], Ahmed M. Sayed[2,3], Rabab Mohammed[2], Hossam M. Hassan[2], Mohamed A. Zaki[2] , Mostafa E. Rateb[2,4,5], Tarek A. Mohammed[6], and Usama Ramadan Abdelmohsen[7*].

[1]*Pharmacognosy Dept., Faculty of Pharmacy, Cairo University, Cairo, Egypt 11787,*

[2]*Pharmacognosy Dept., Faculty of Pharmacy, Beni-Suef University, Beni-Suef, Egypt 62514,*

[3]*Pharmacognosy Dept., Faculty of Pharmacy, Nahda University, Beni-Suef, Egypt 62513,*

[4]*Marine Biodiscovery Centre, University of Aberdeen, Scotland, UK, AB24 3UE,*

[5]*School of Computing, Engineering and Physical Sciences, University of the West of Scotland, Paisley PA1 2BE, UK,*

[6]*Marine Invertebrates, National Institute of Oceanography and Fisheries, Red Sea Branch, Hurghada, Egypt 84511,*

[7]*Department of Pharmacognosy, Faculty of Pharmacy, Minia University, Minia, Egypt 61519.*

*Correspondence: Usama Ramadan Abdelmohsen, ramadanusama80@gmail.com, +201111595772.

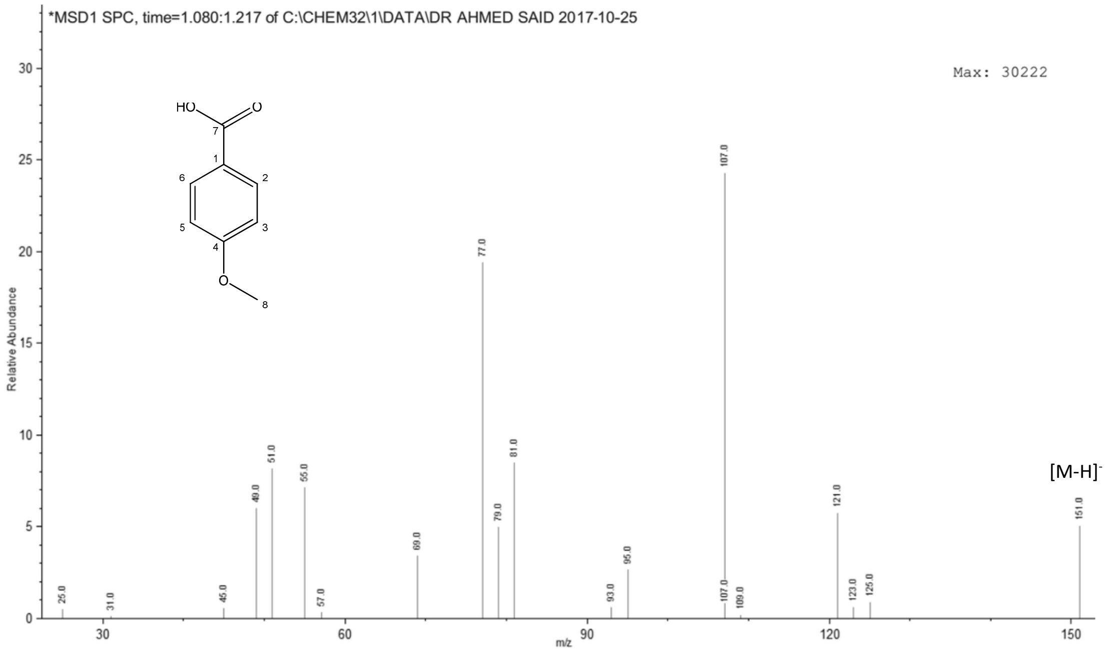

Fig. S1. ESI-MS spectrum of *p*-anisic acid (1)

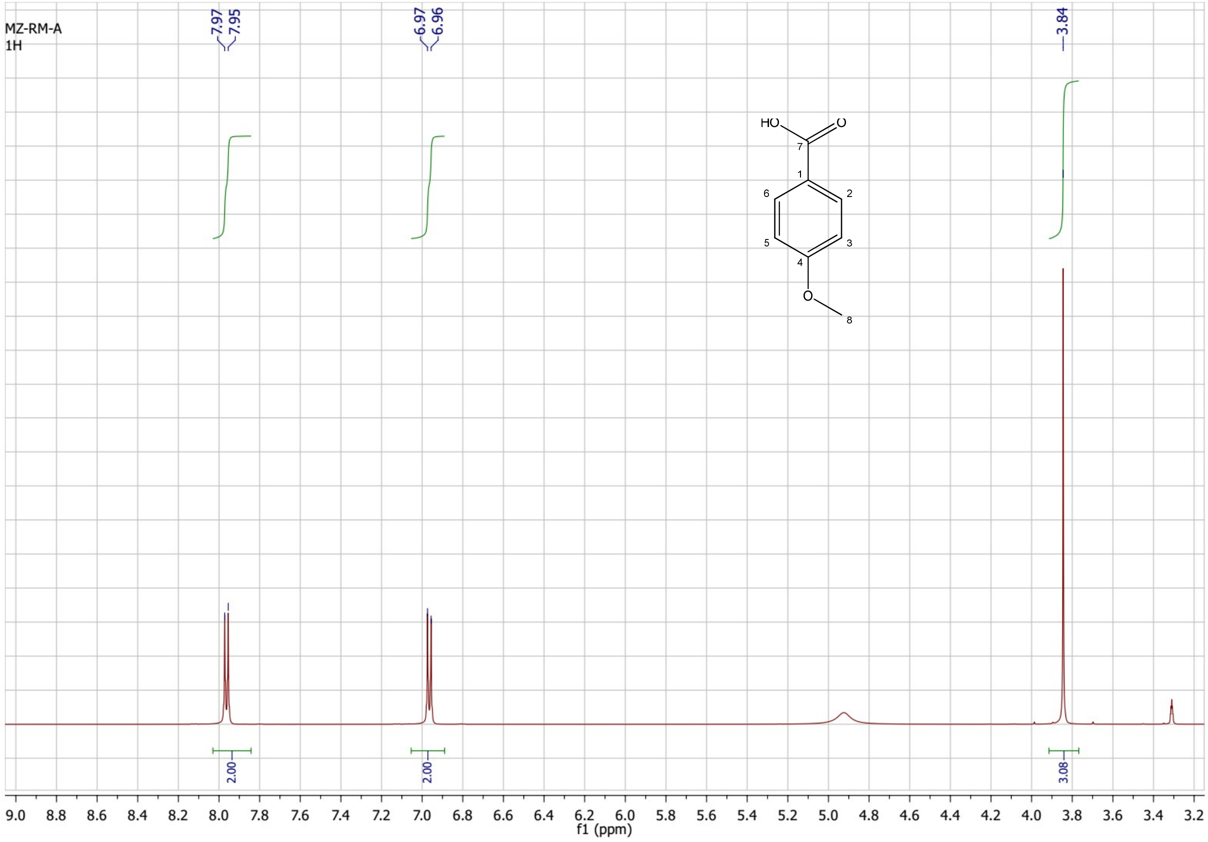

Fig. S2. ^{1}H NMR spectrum of *p*-anisic acid (1) in CD_3OD.

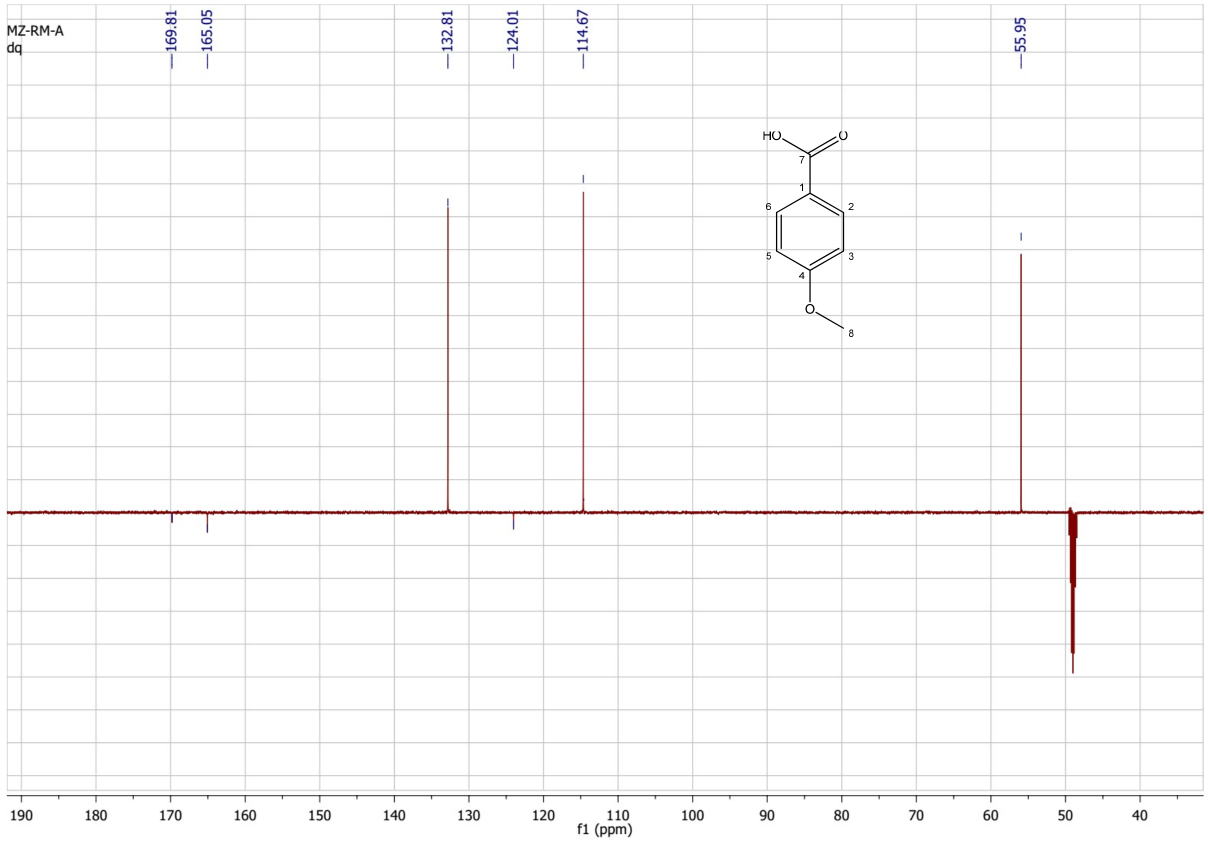

Fig. S3. DEPTQ spectrum of *p*-anisic acid (1) in CD_3OD.

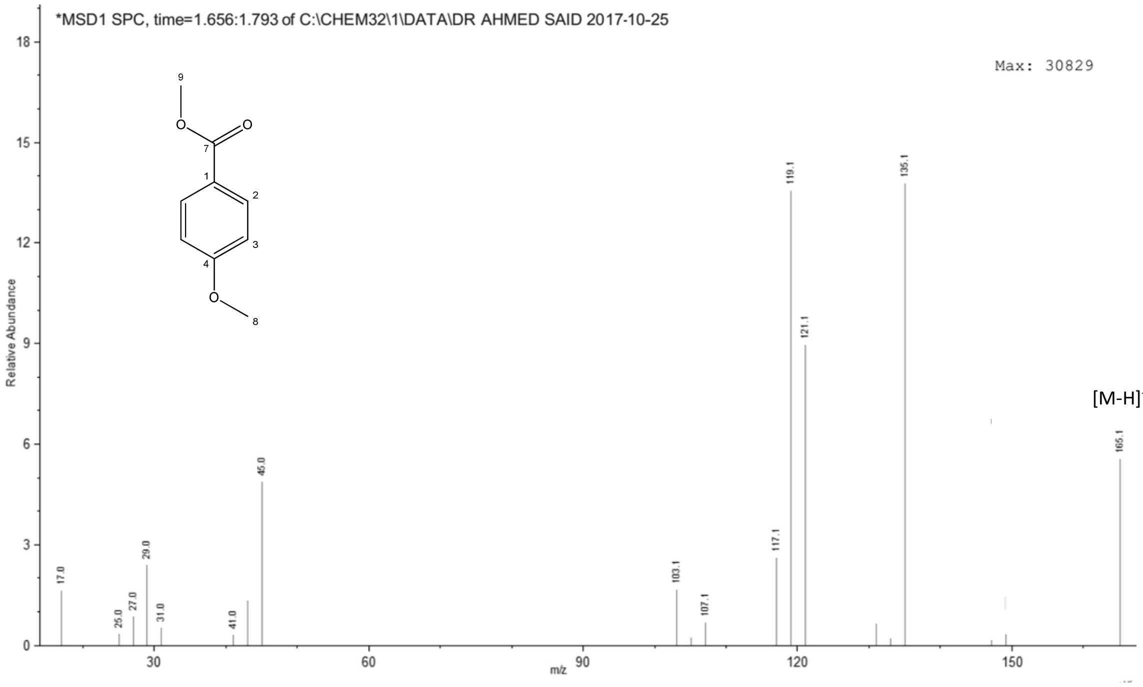

Fig. S4. ESI-MS spectrum of *p*-anisic acid methyl ester (2)

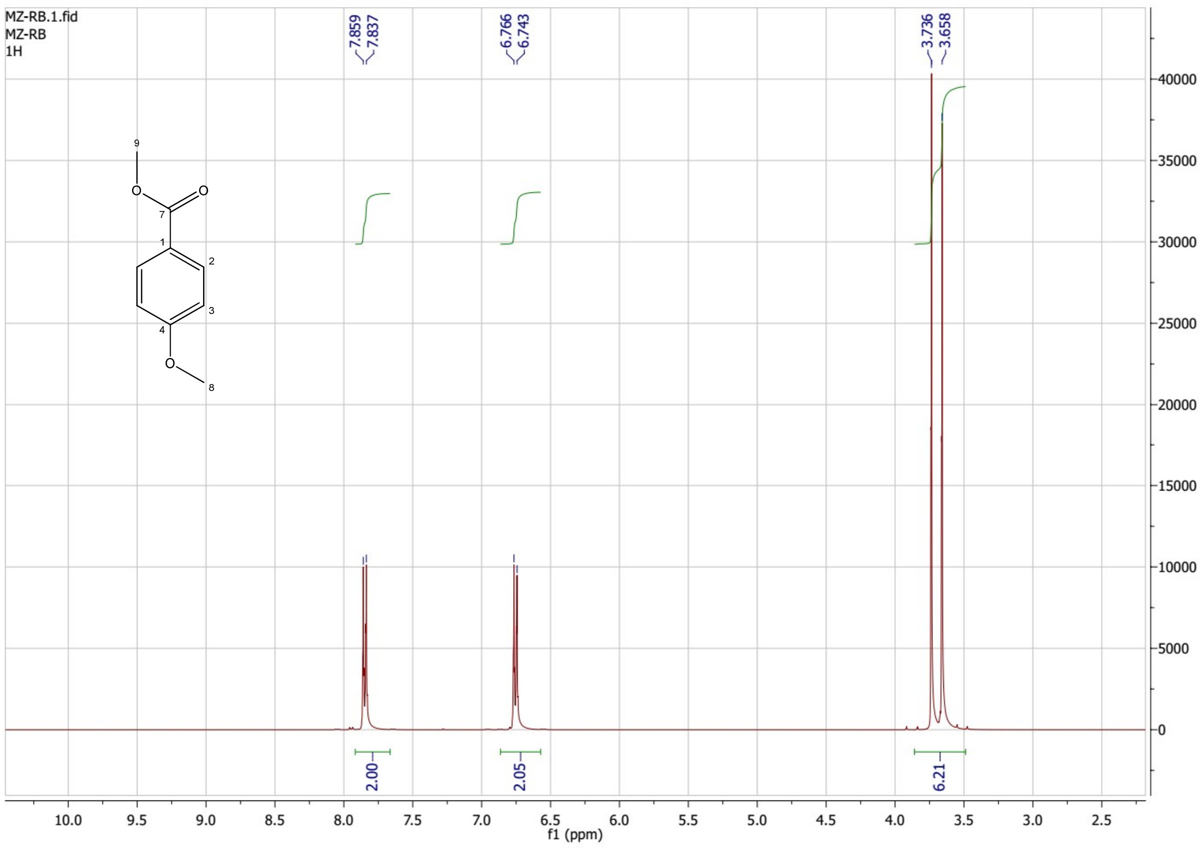

Fig. S5. ^{1}H NMR spectrum of *p*-anisic acid methyl ester (2) in $CDCl_3$

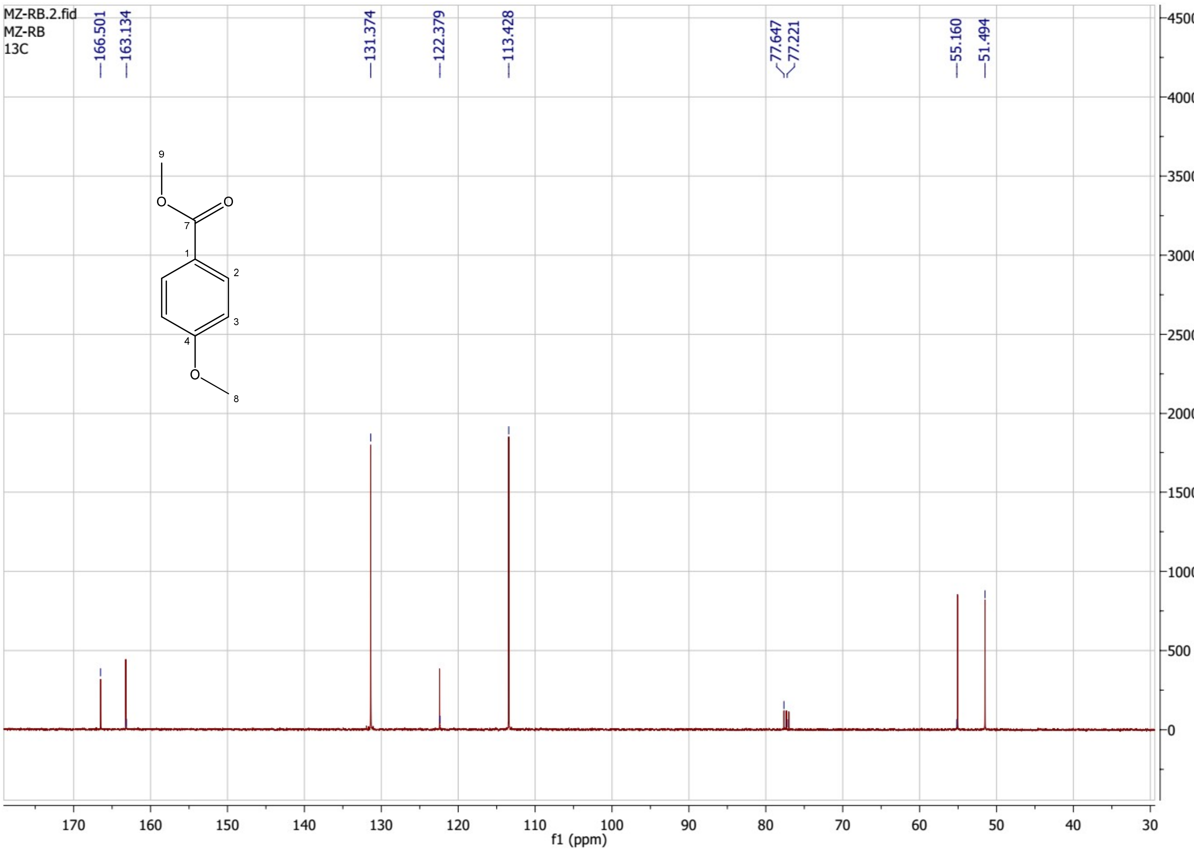

Fig. S6. ^{13}C NMR spectrum of *p*-anisic acid methyl ester (2) in $CDCl_3$

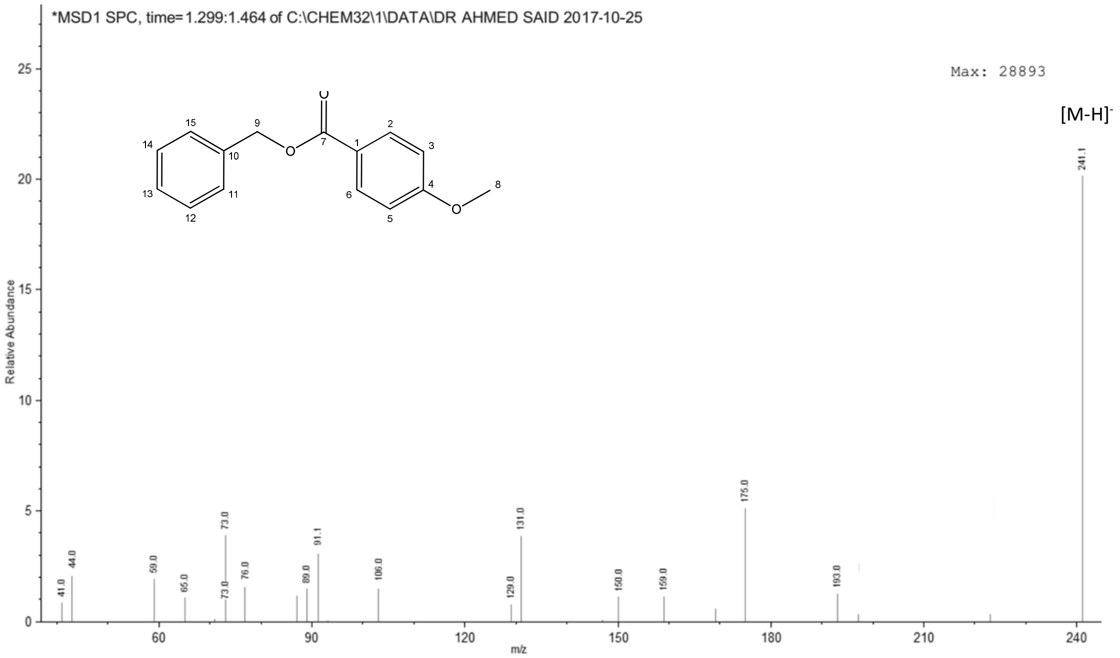

Fig. S7. ESI-MS spectrum of benzyl anisate (3)

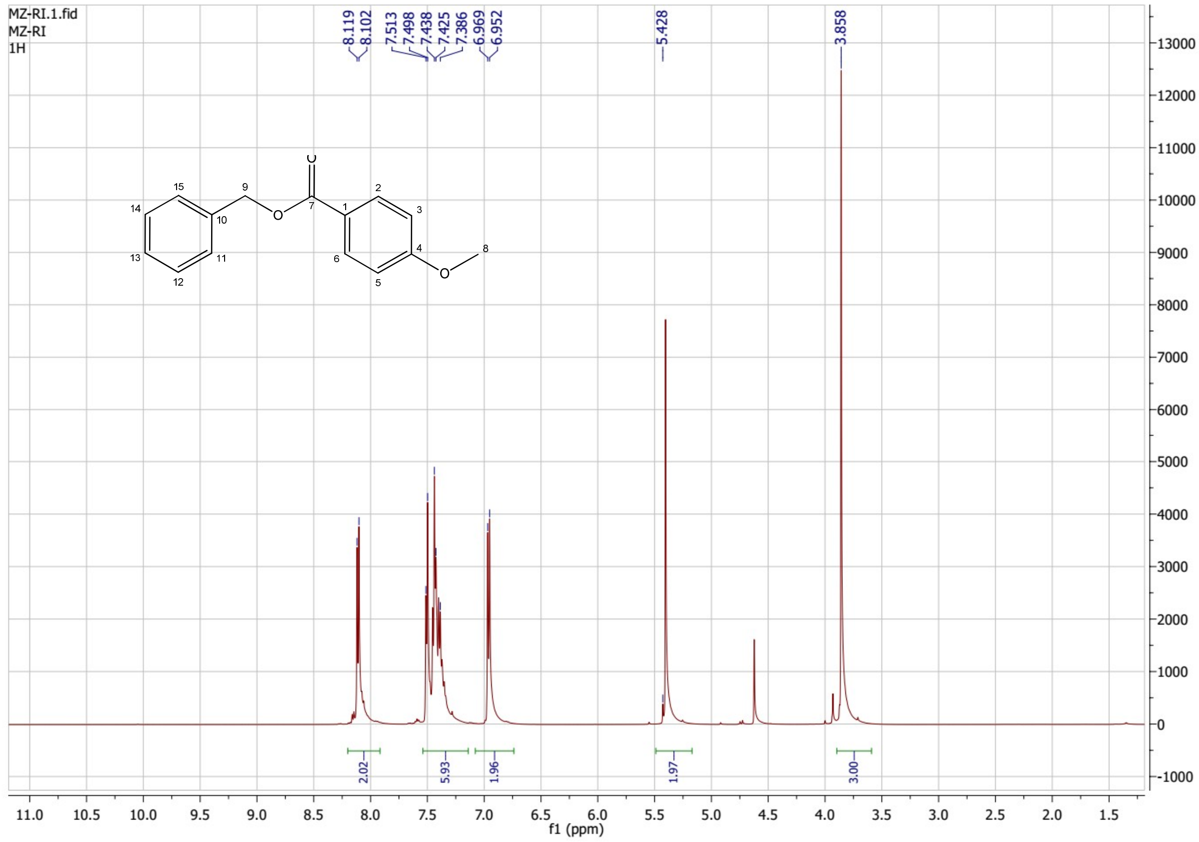

Fig. S8. ^{1}H NMR spectrum of benzyl anisate (3) in $CDCl_3$

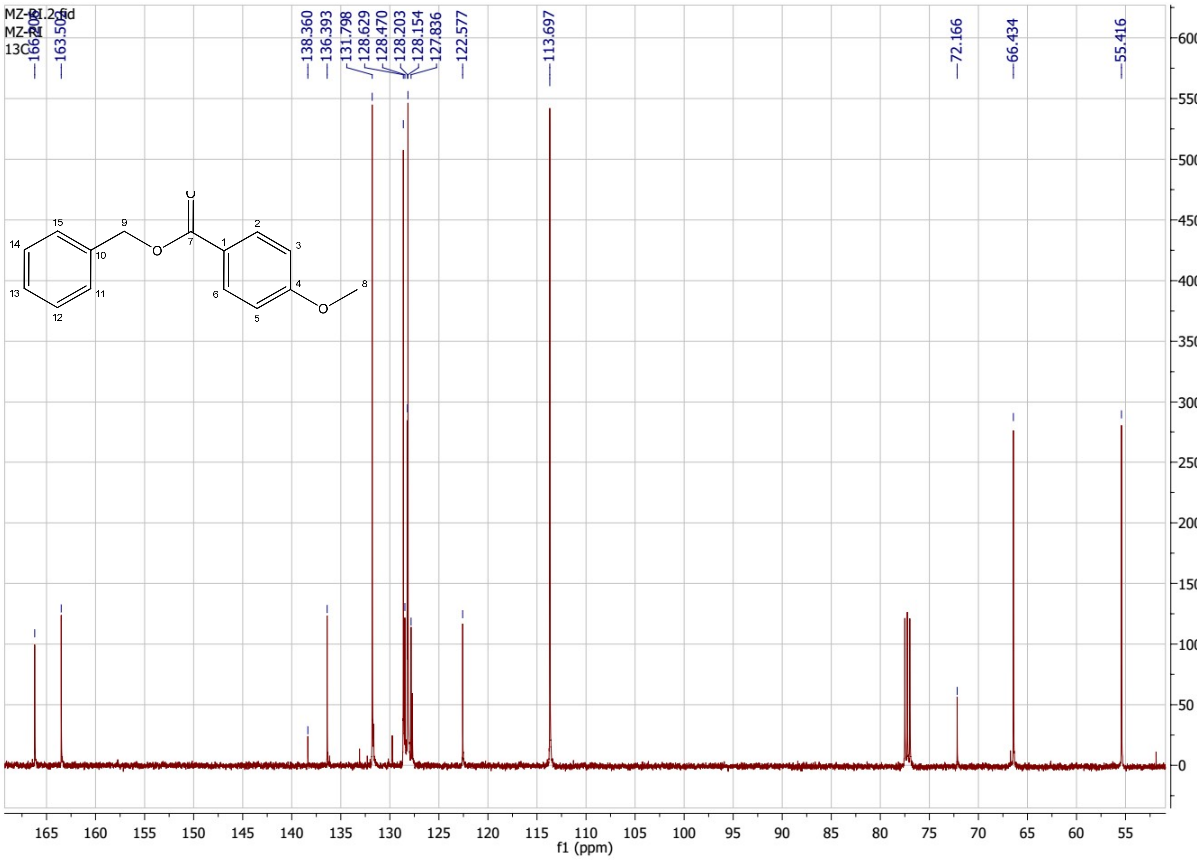

Fig. S9. ^{13}C NMR spectrum of benzyl anisate (3) in $CDCl_3$

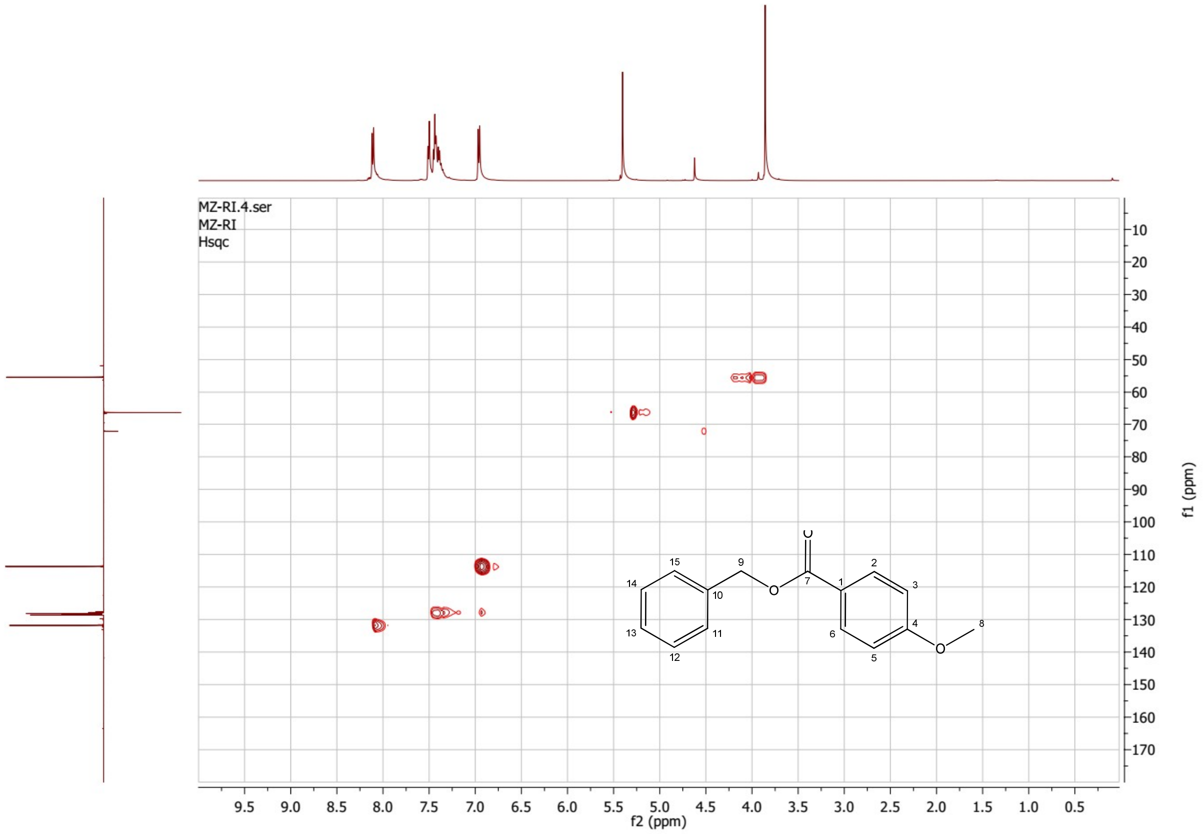

Fig. S10. HSQC spectrum of benzyl anisate (3)

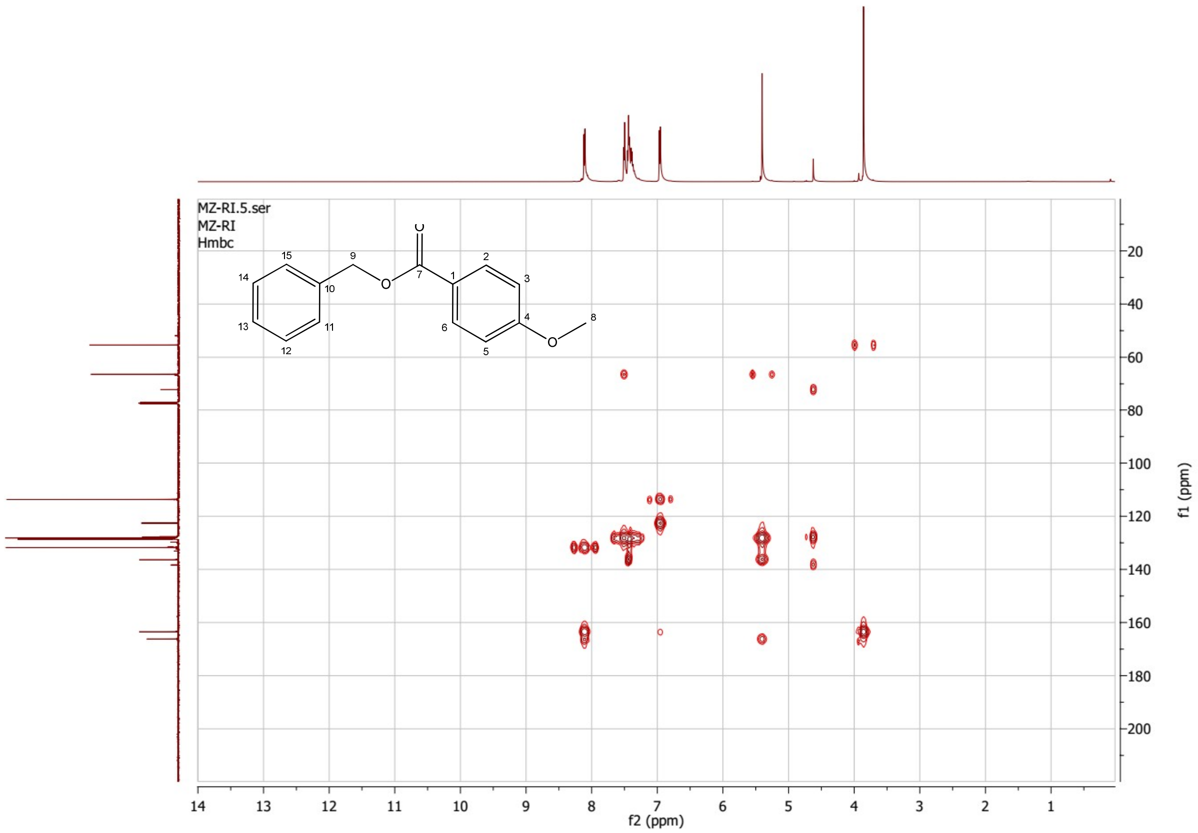

Fig. S11. HMBC spectrum of benzyl anisate (3)

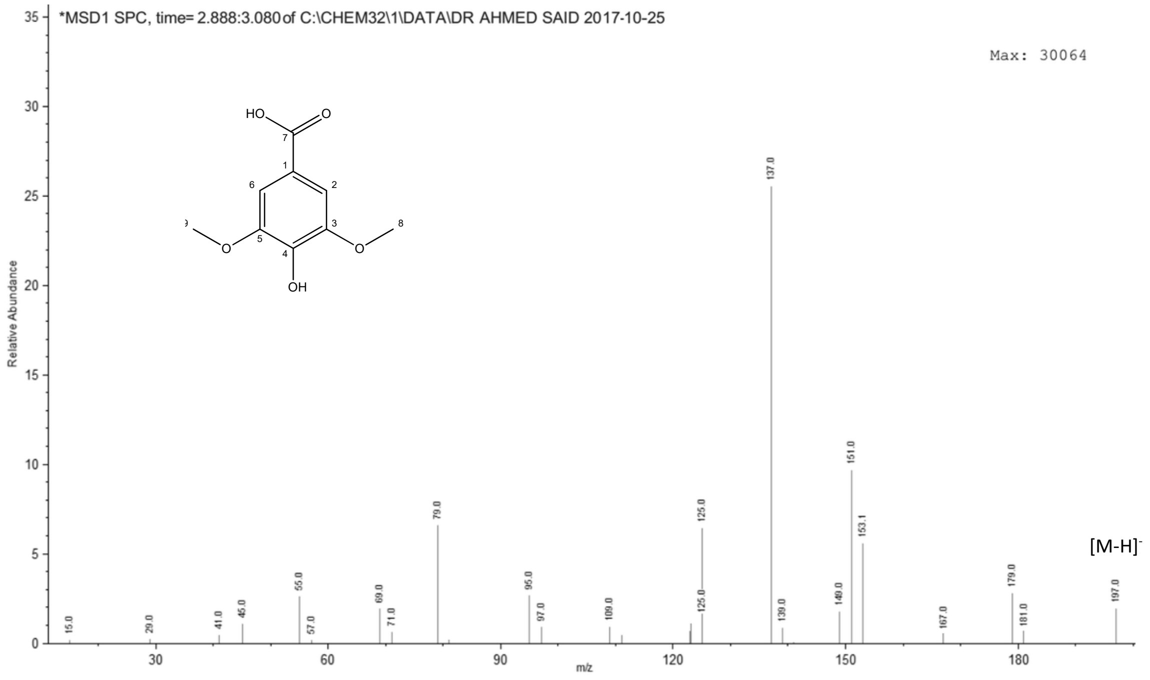

Fig. S12. ESI-MS spectrum of syringic acid (4)

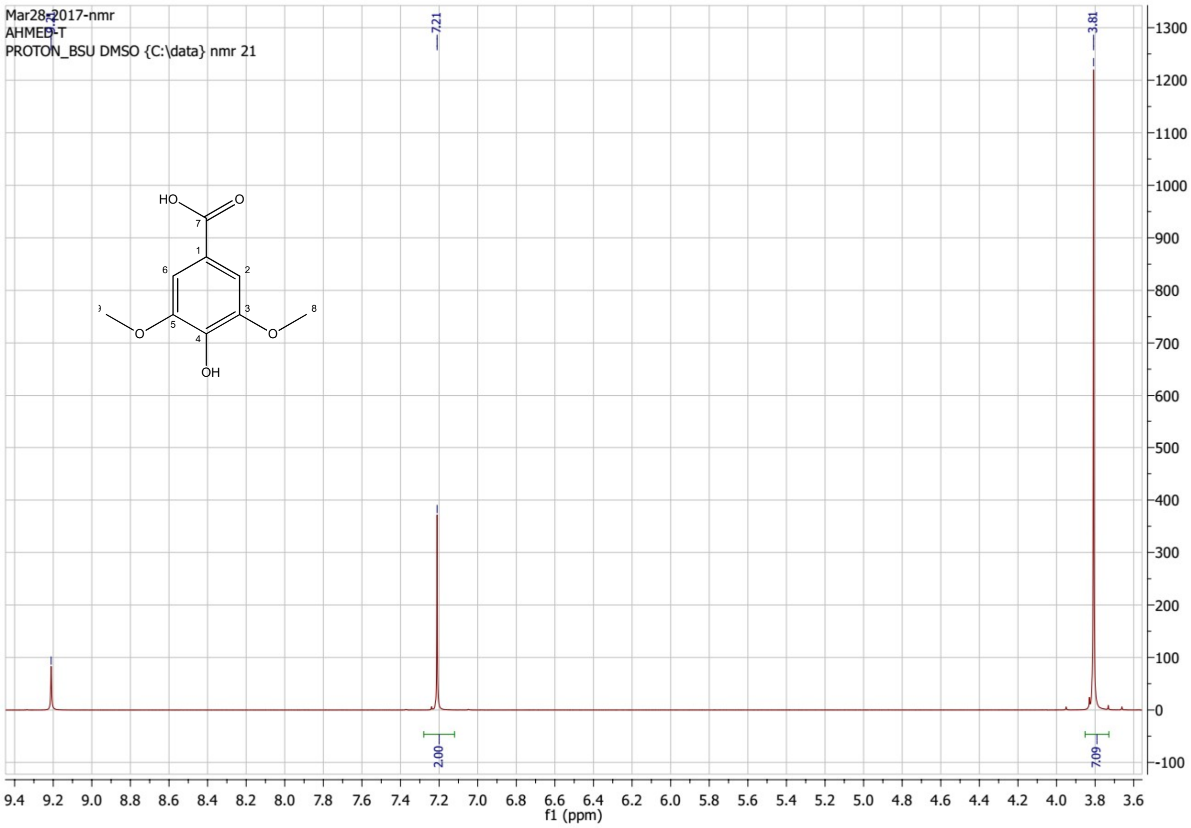

Fig. S13. ^{1}H NMR spectrum of syringic acid (4) in DMSO-*d₆*

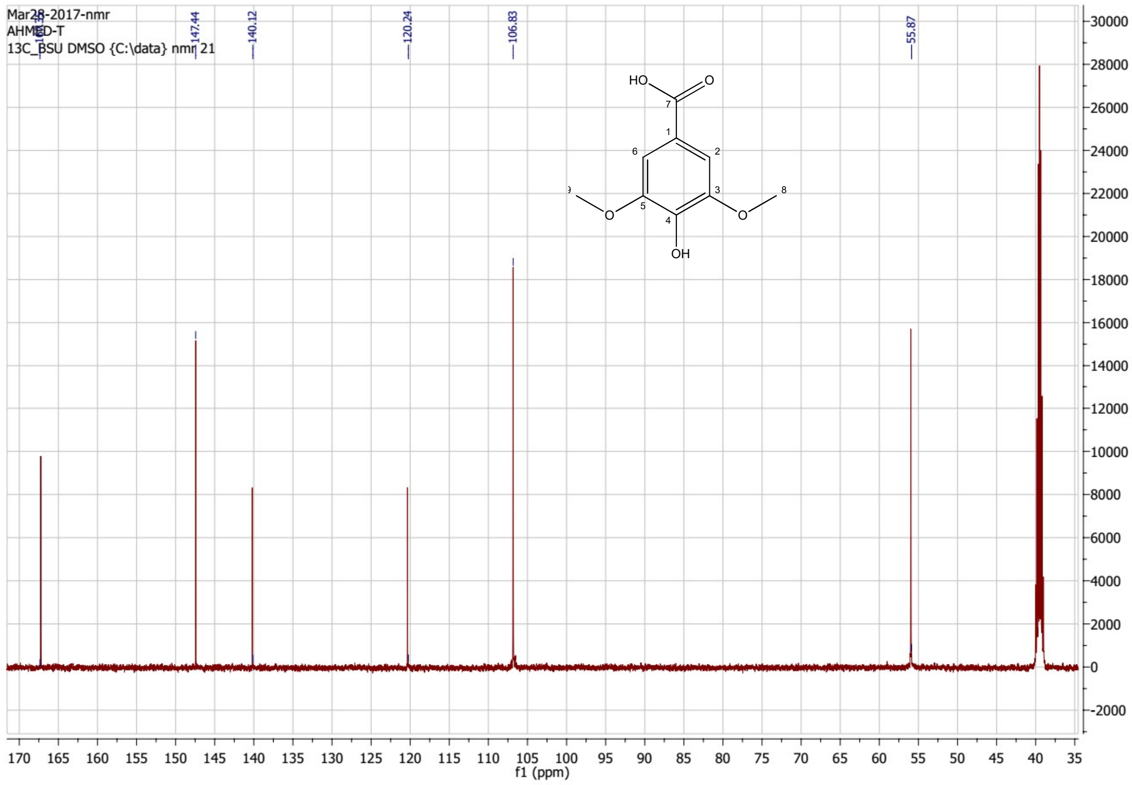

Fig. S14. ^{13}C NMR spectrum of syringic acid (4) in DMSO-d_6

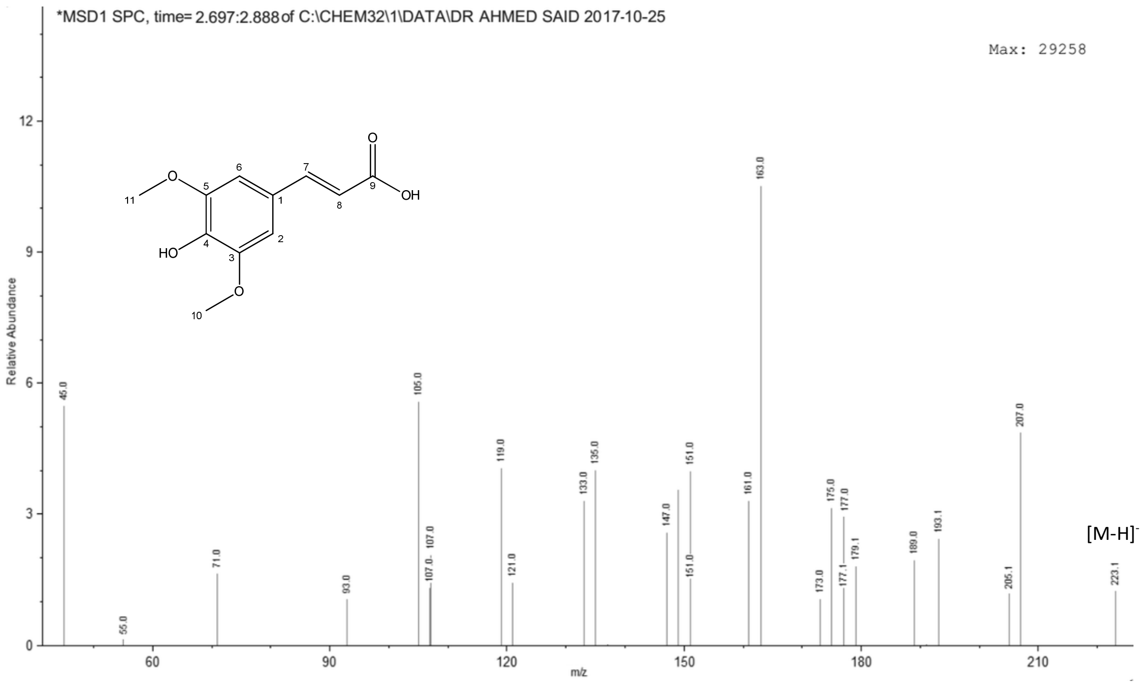

Fig. S15. ESI-MS spectrum of sinapic acid (5)

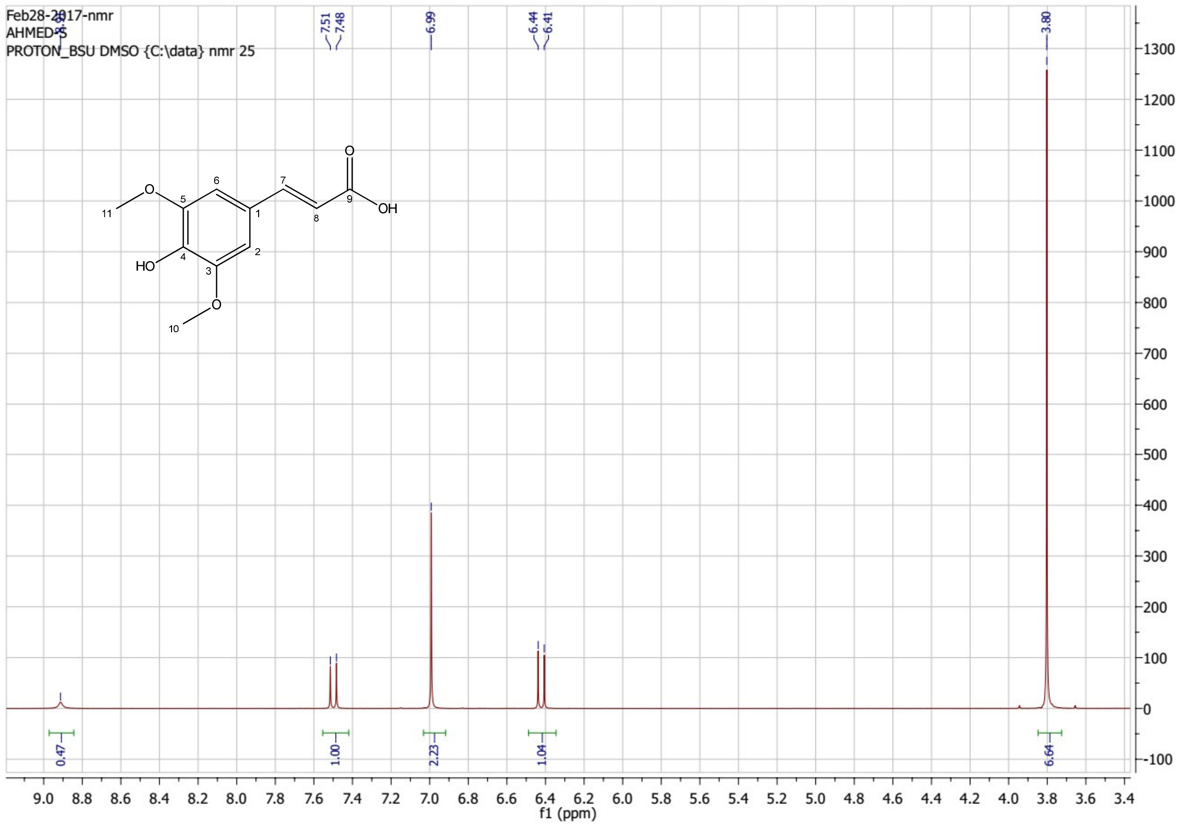

Fig. S16. ^{1}H NMR spectrum of sinapic acid (5) in DMSO-d_6

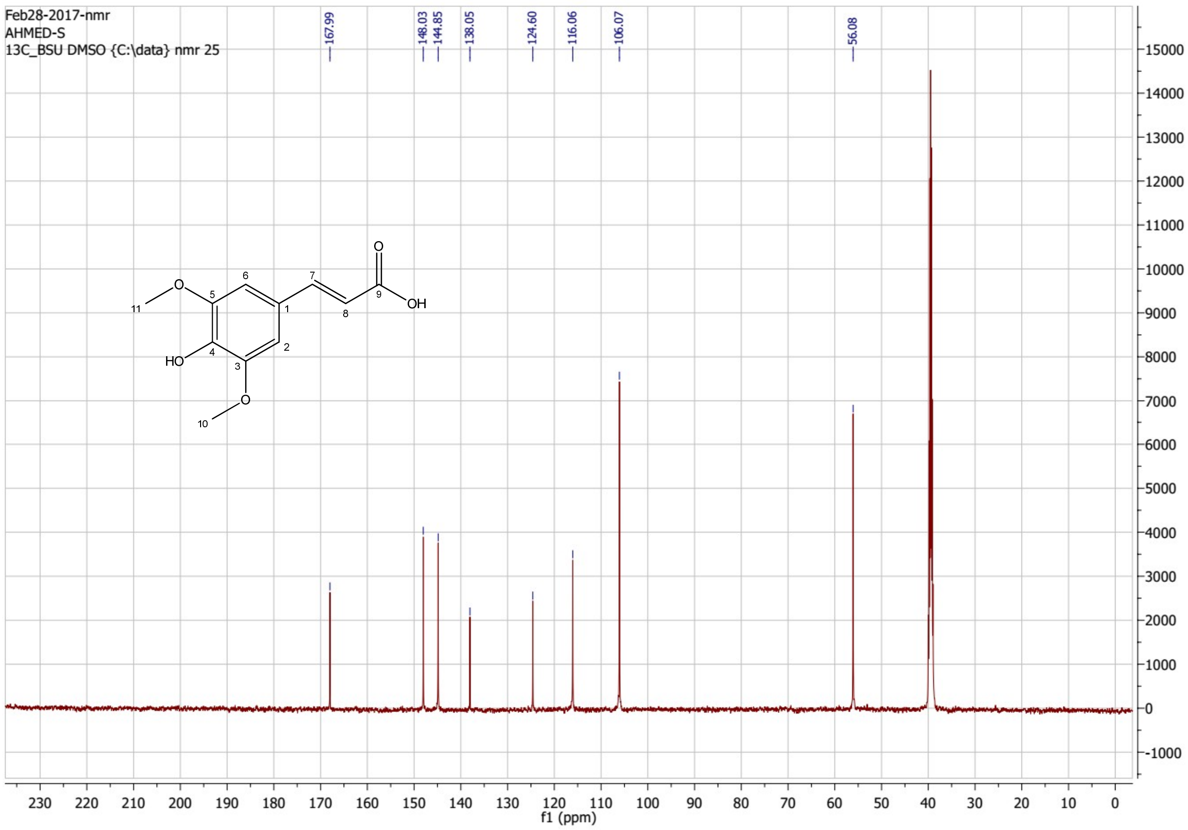

Fig. S17. ^{13}C NMR spectrum of of sinapic acid (5) in DMSO-*d_6*

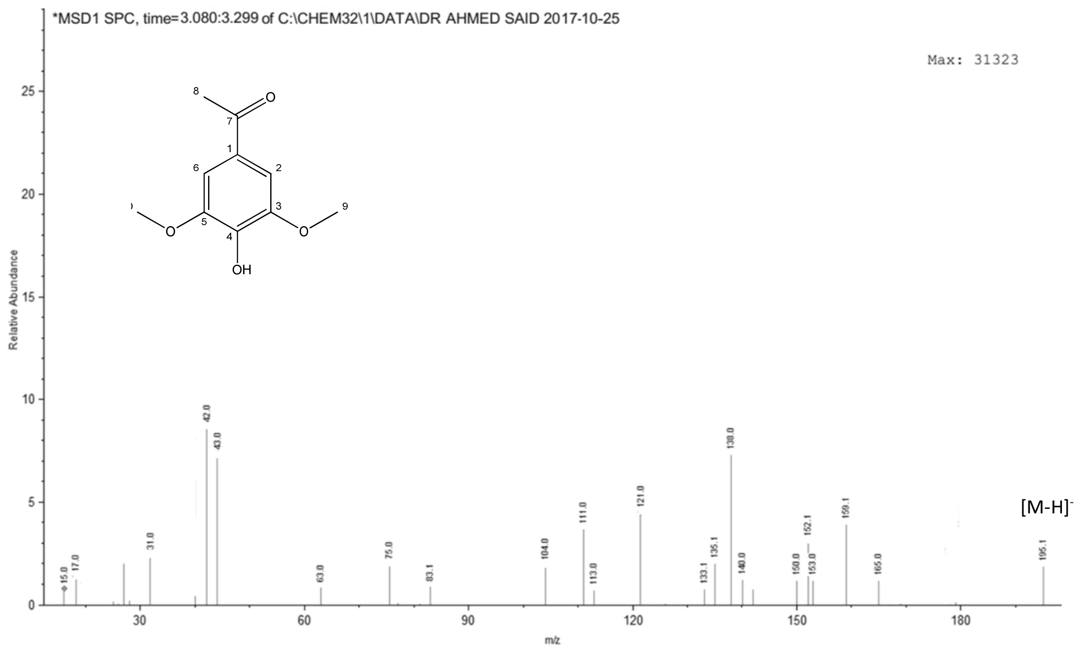

Fig. S18. ESI-MS spectrum of acetosyringone (6)

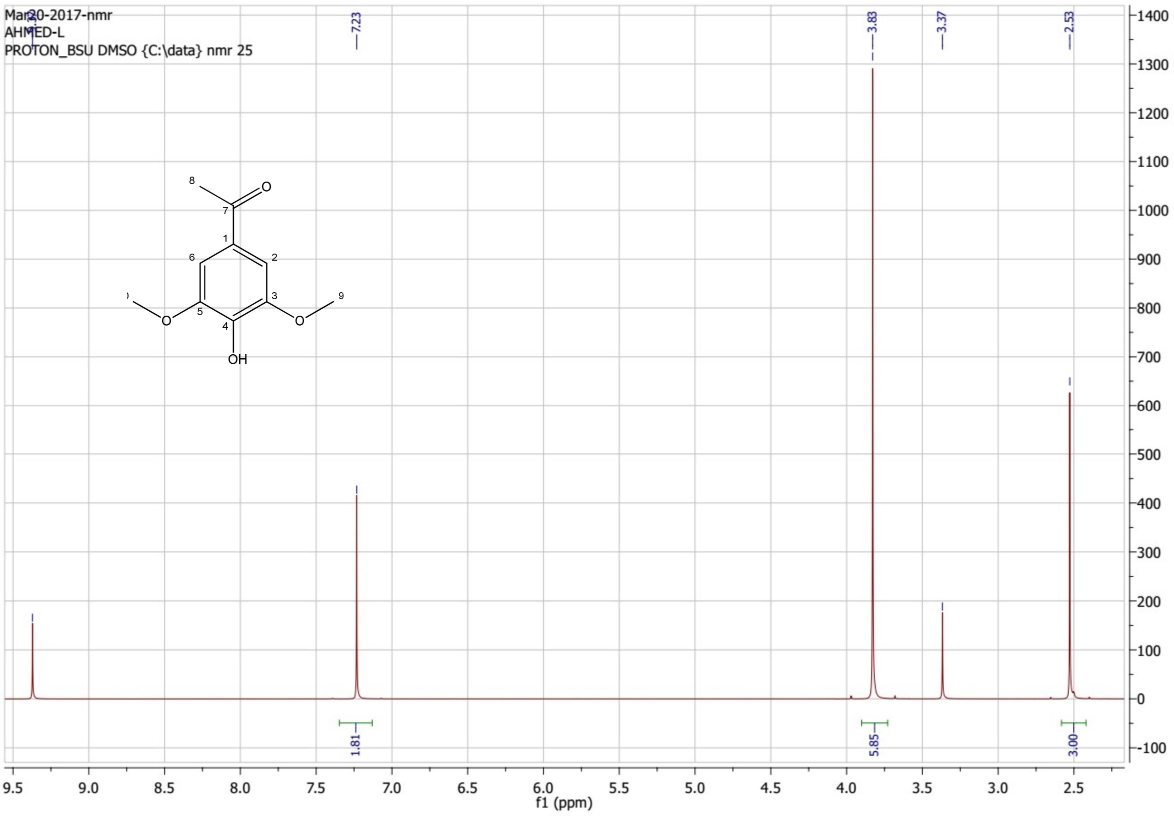

Fig. S19. ^{1}H NMR spectrum of acetosyringone (6) in DMSO-d_6

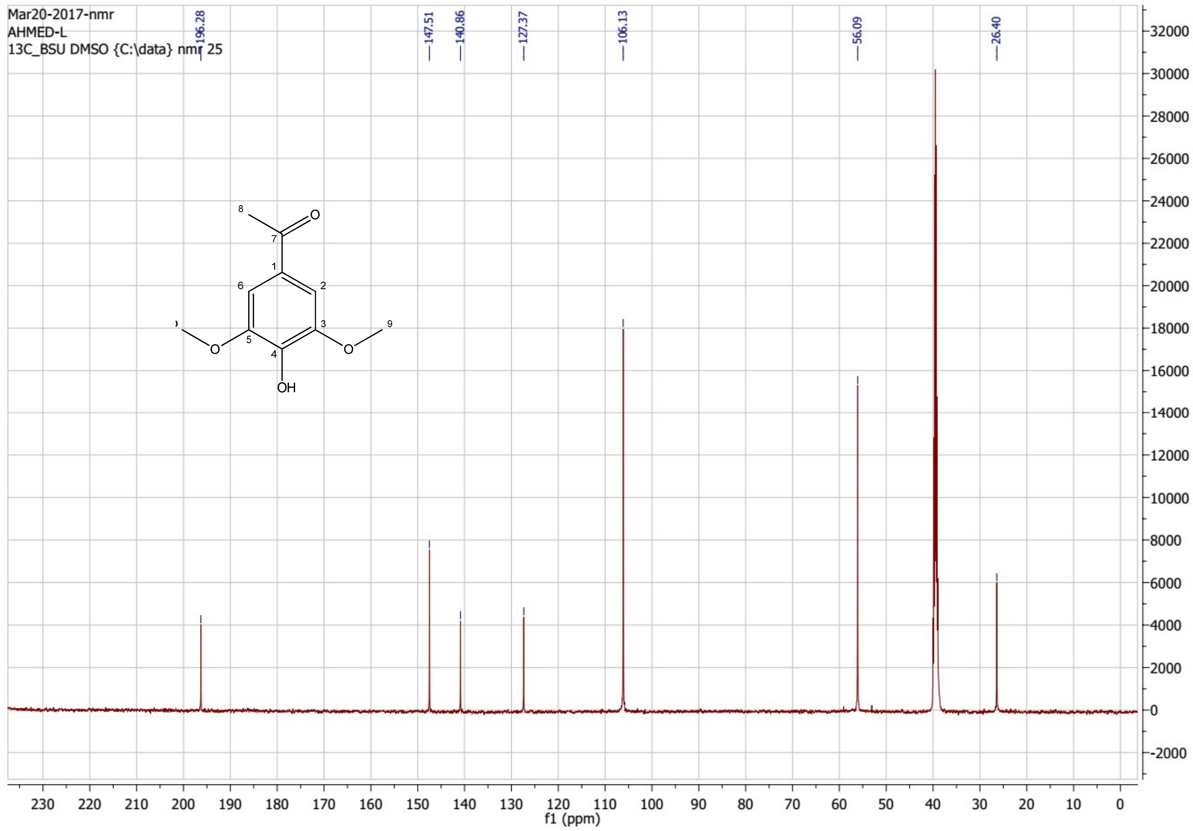

Fig. S20. ^{13}C NMR spectrum of acetosyringone (6) in DMSO-d_6

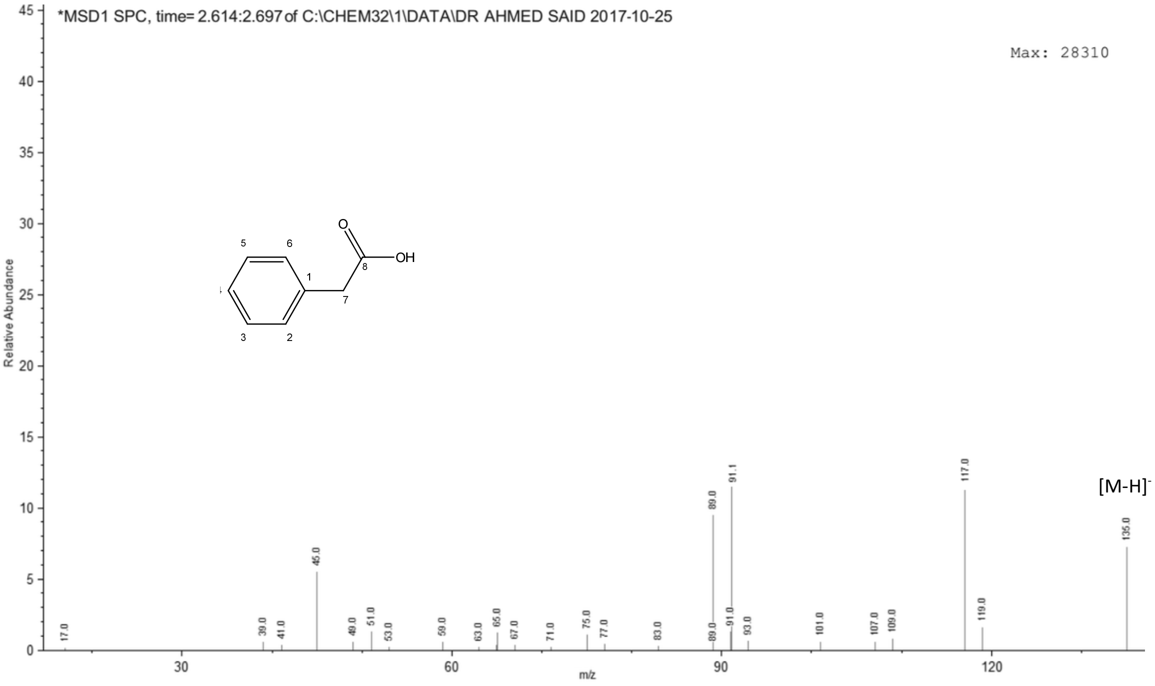

Fig. S21. ESI-MS spectrum of phenyl acetic acid (7)

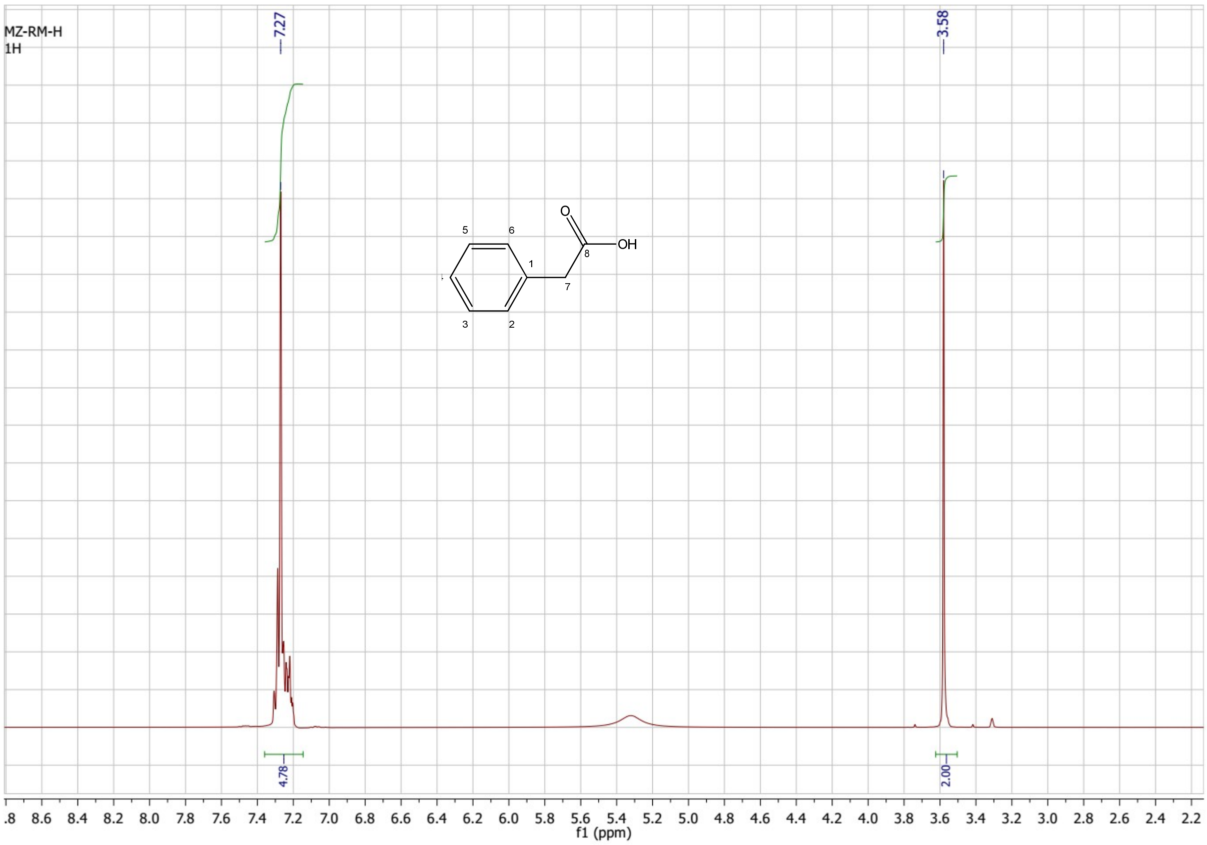

Fig. S22. ¹H NMR spectrum of phenyl acetic acid (7) in CD_3OD

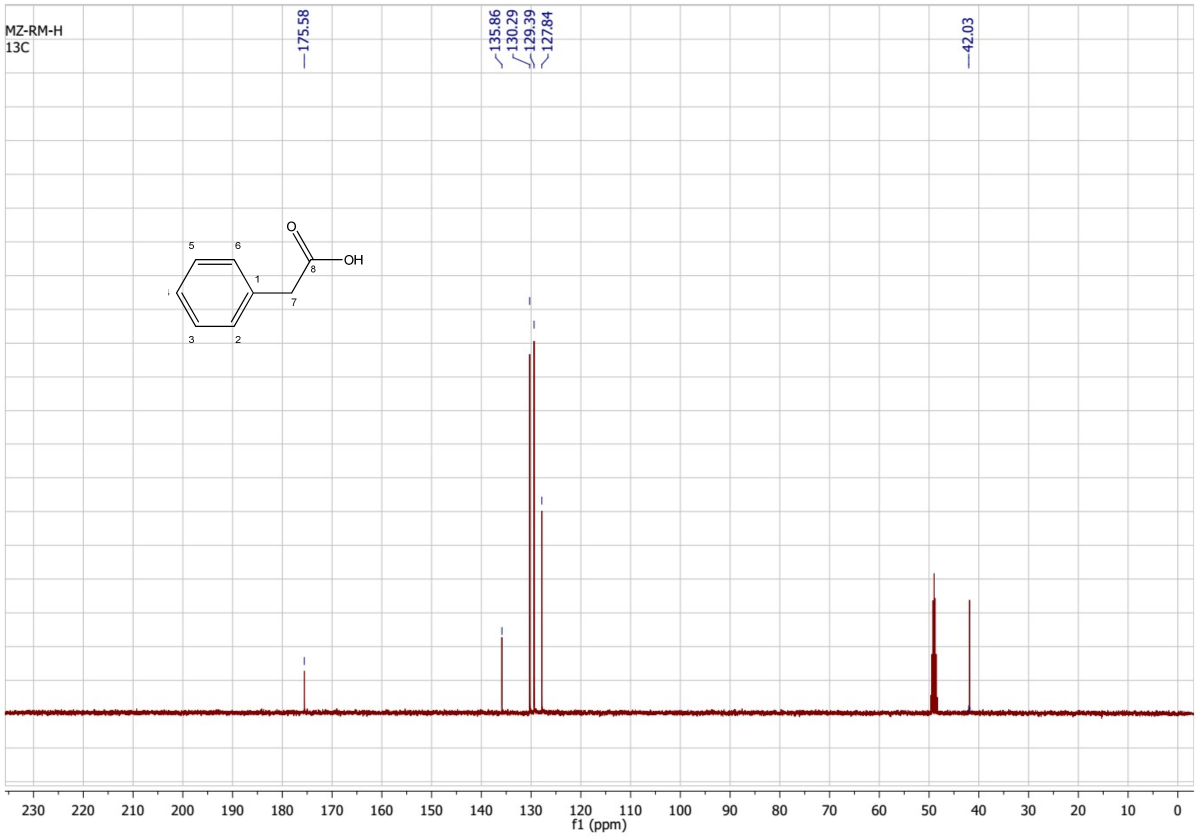

Fig. S23. ^{13}C NMR spectrum of phenyl acetic acid (7) in CD_3OD

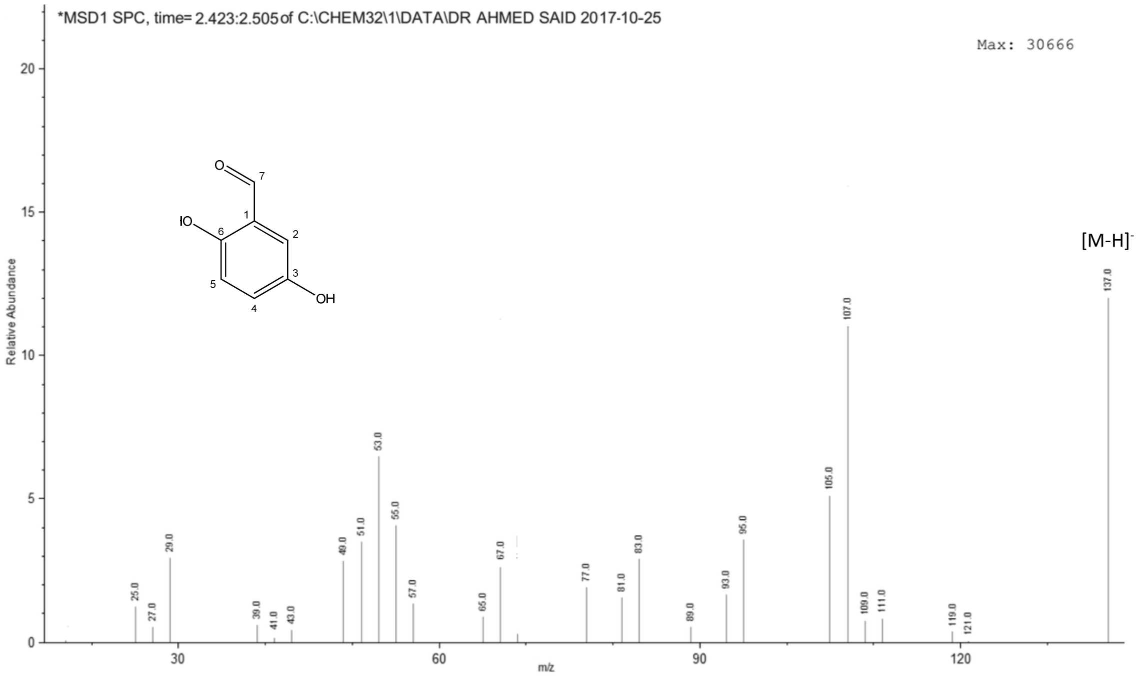

Fig. S24. ESI-MS spectrum of gentisaldehyde (8)

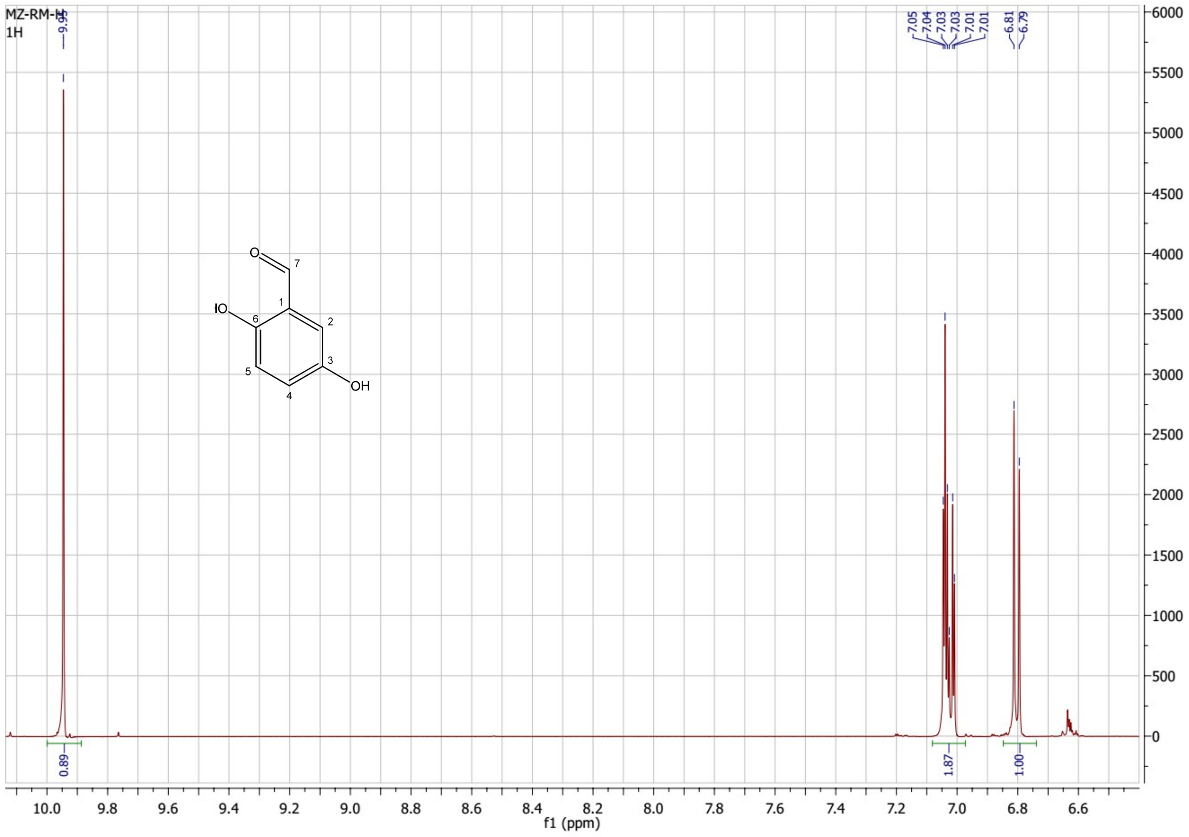

Fig. S25. **^{1}H NMR spectrum of gentisaldehyde (8) in CD_3OD**

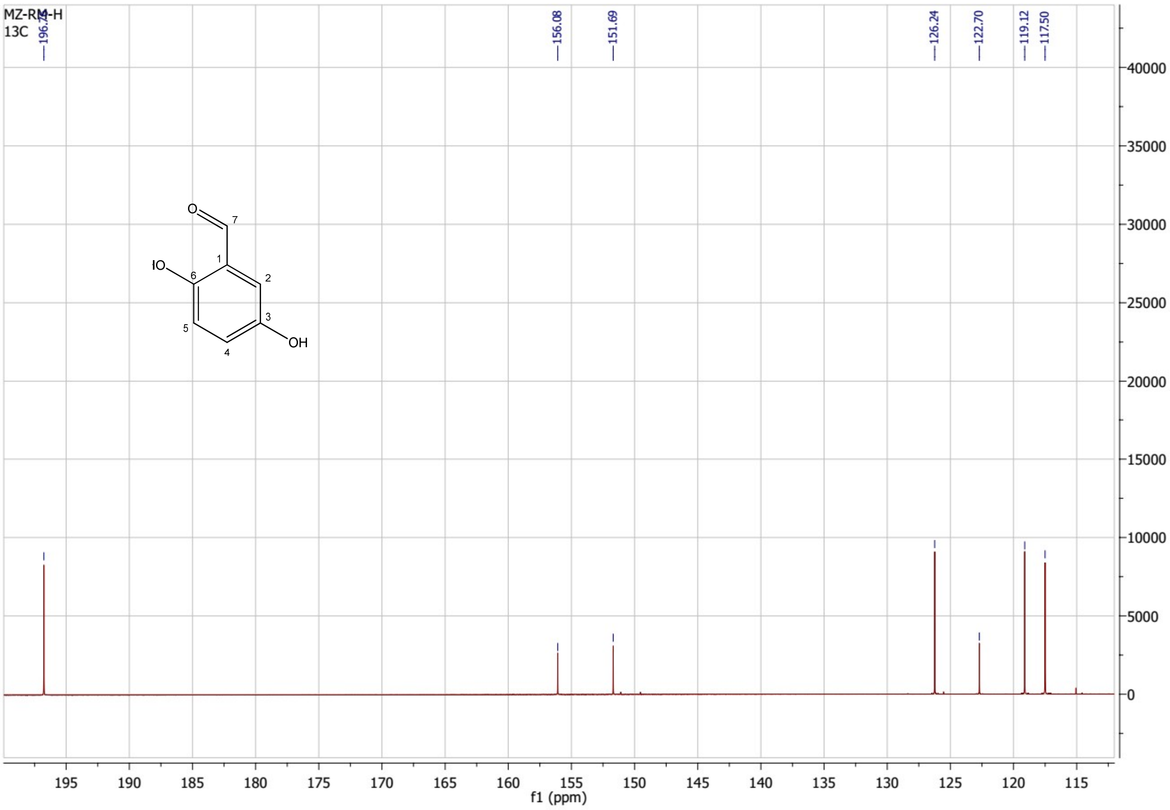

Fig. S26. ^{13}C NMR spectrum of gentisaldehyde (8) in CD_3OD

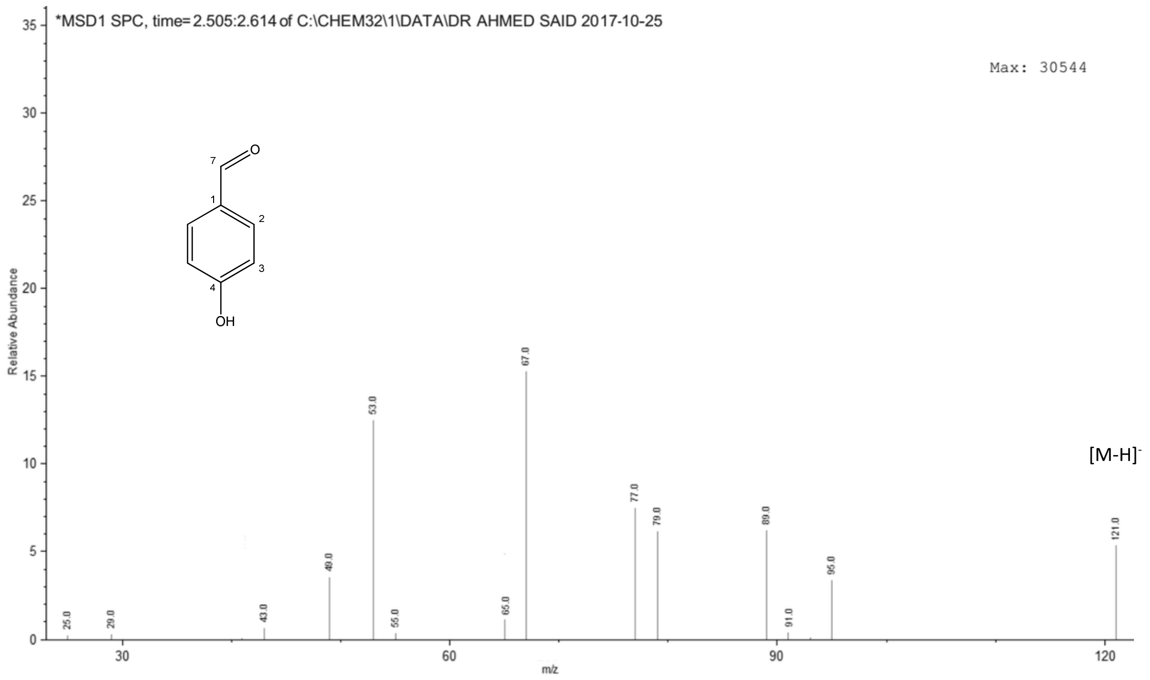

Fig. S27. ESI-MS spectrum of *p*-hydroxy benzaldehyde (9)

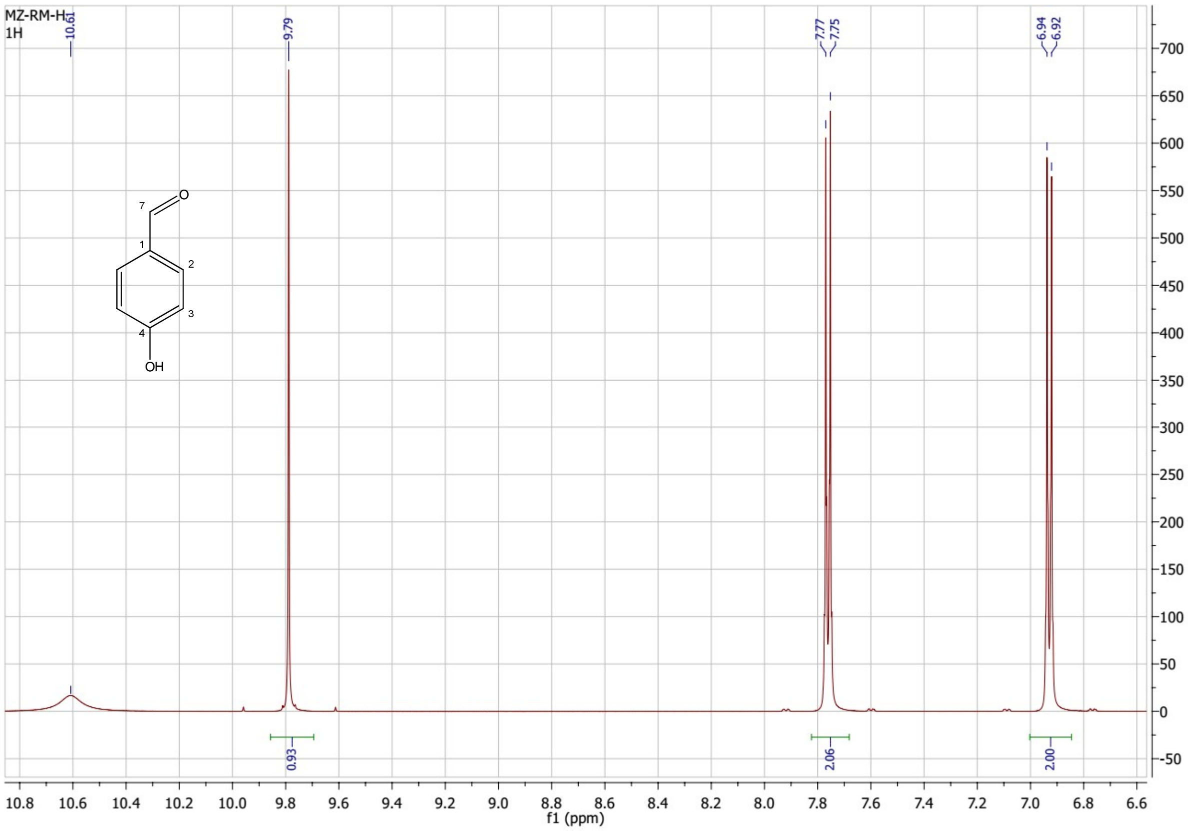

Fig. S28. [1]**H NMR spectrum of *p*-hydroxy benzaldehyde (9) in DMSO-d_6**

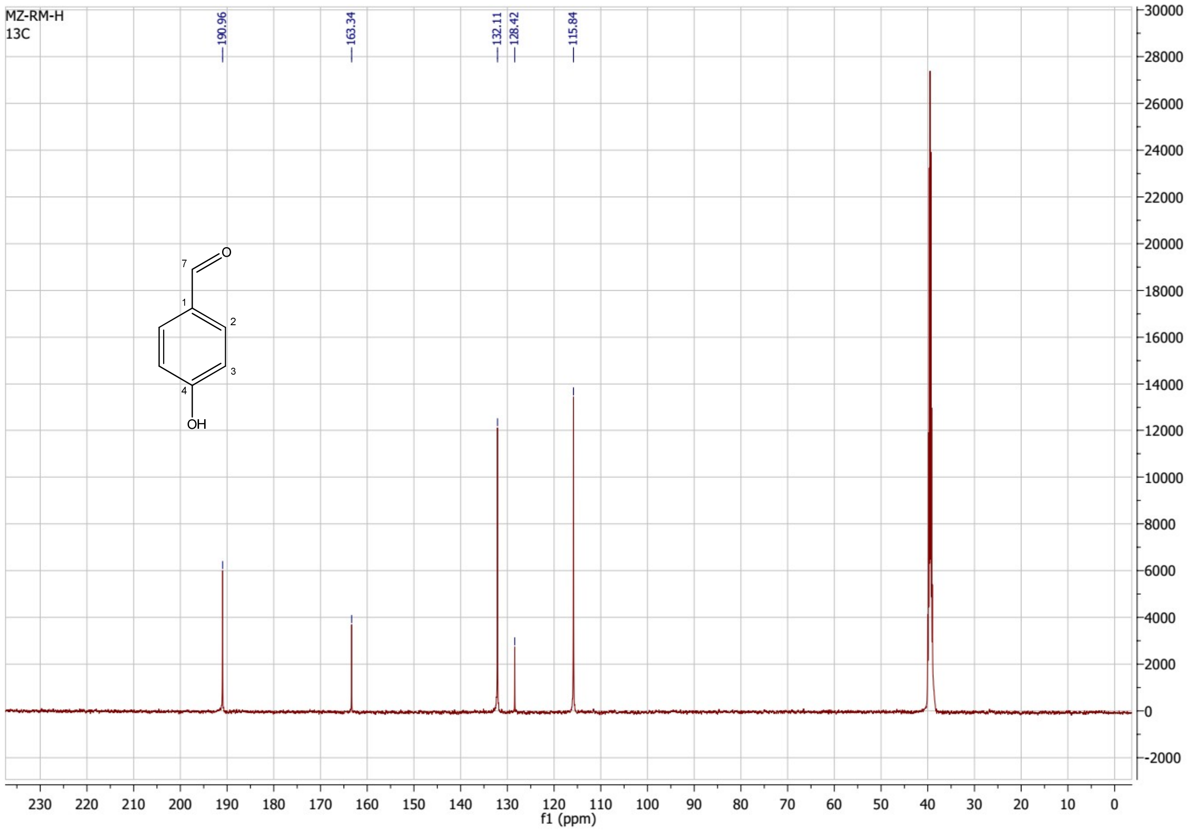

Fig. S29. ^{13}**C NMR spectrum of** ***p*****-hydroxy benzaldehyde (9) in DMSO-*d*$_6$**

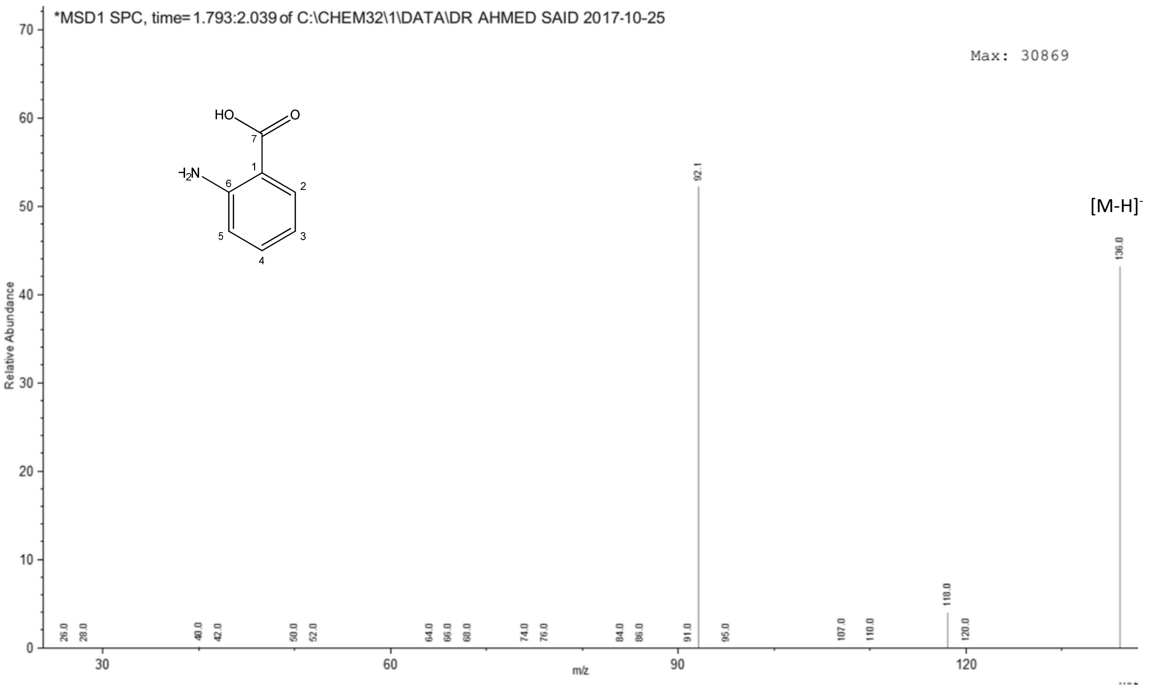

Fig. S30. ESI-MS spectrum of anthranilc acid (10)

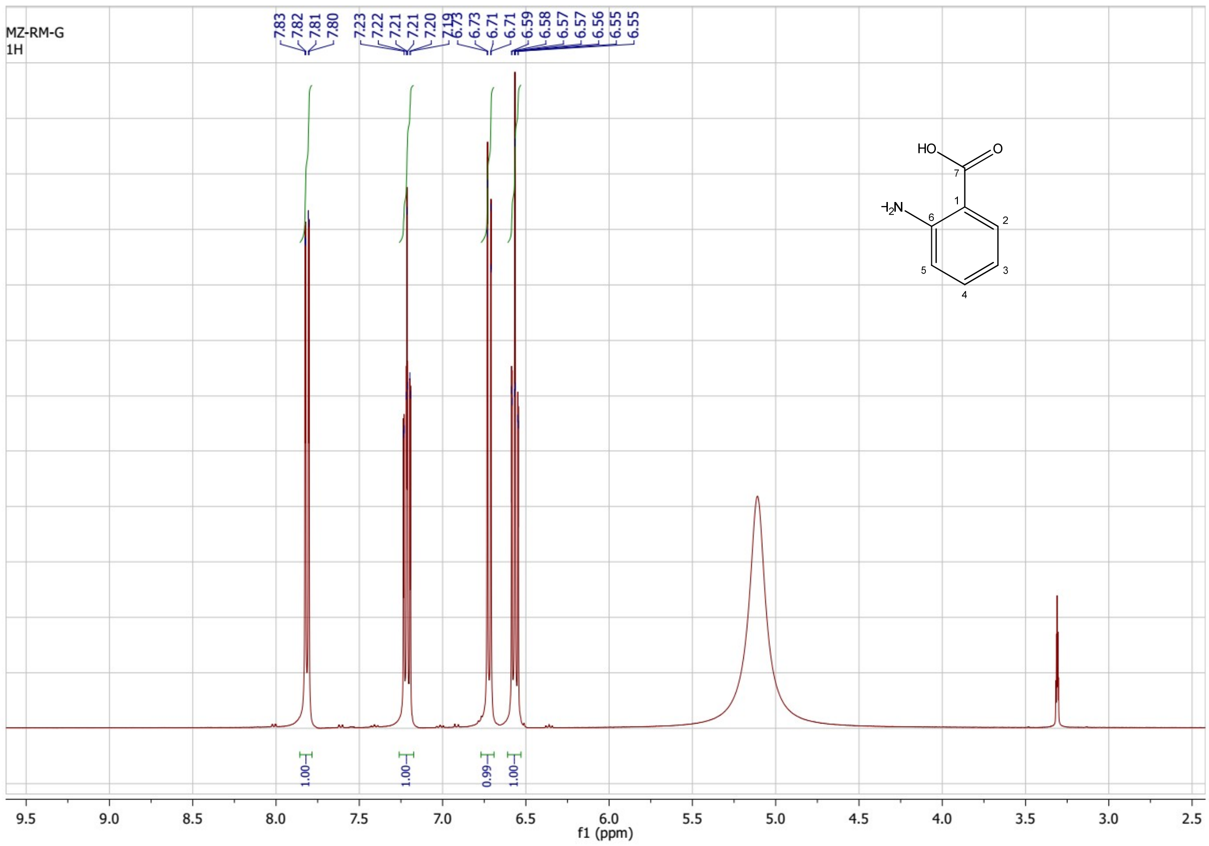

Fig. S31. **^{1}H NMR spectrum of anthranilc acid (10) in CD_3OD**

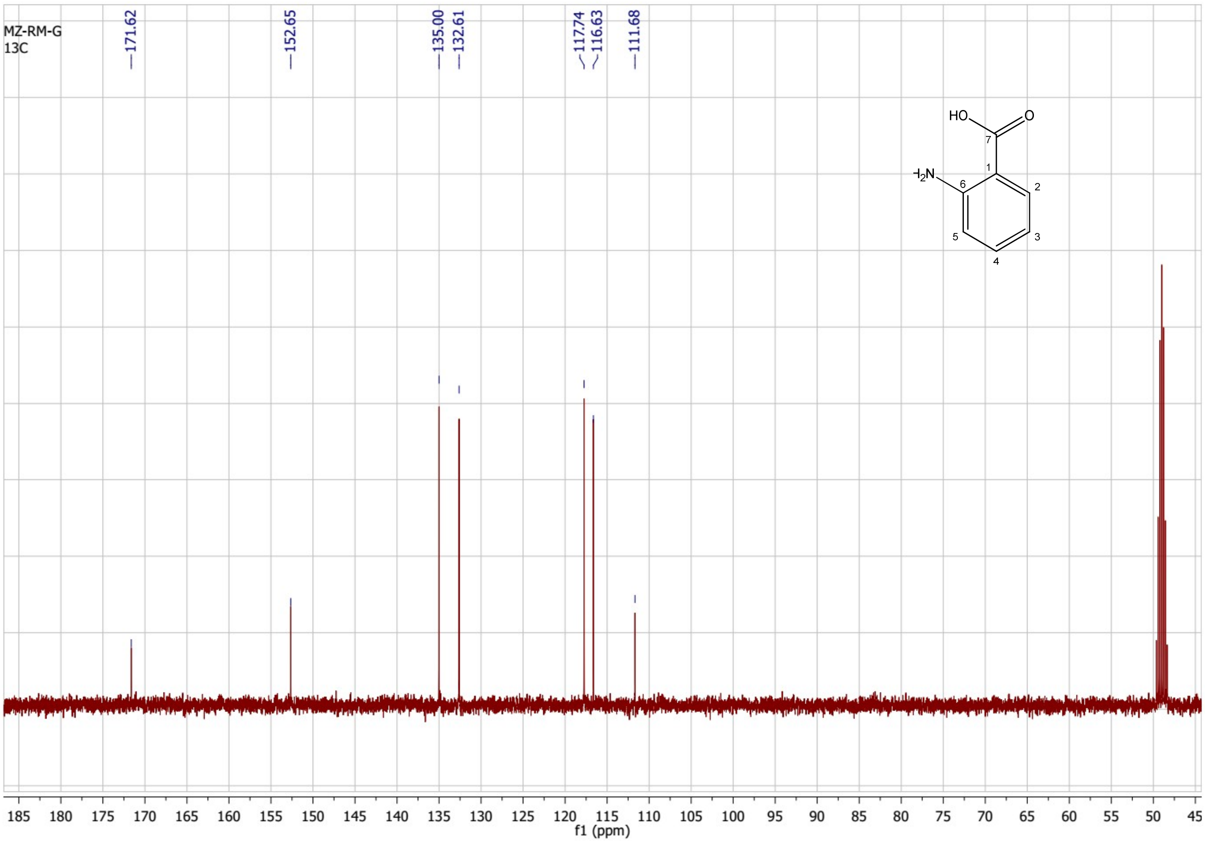

Fig. S32. ^{13}C NMR spectrum of anthranilic acid (10) in CD_3OD

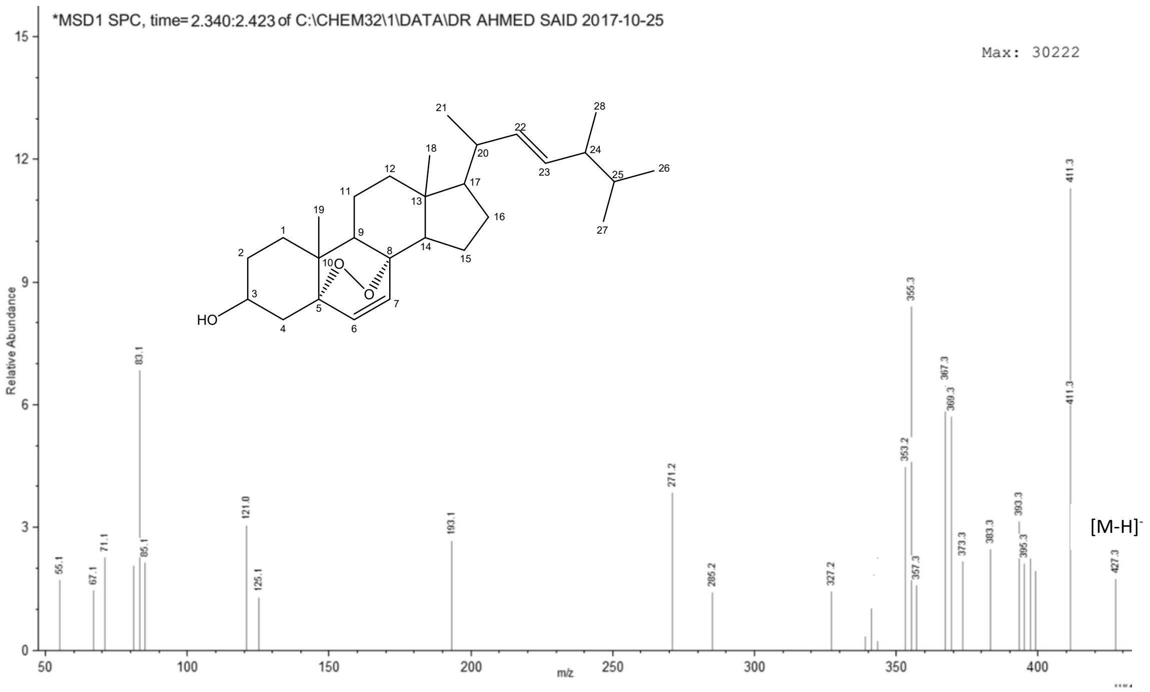

Fig. S33. ESI-MS spectrum of ergosterol peroxide (11)

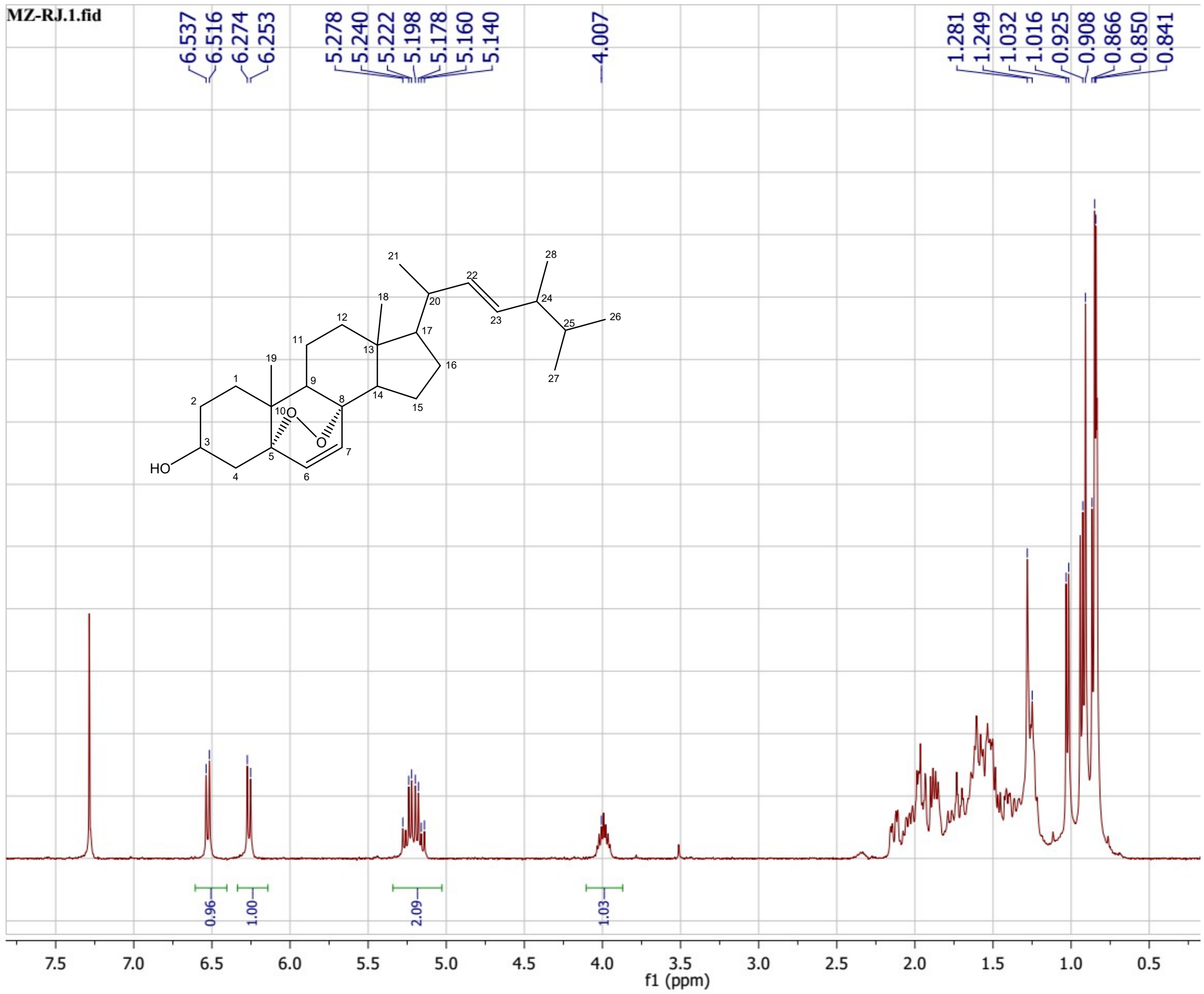

Fig. S34. ^{1}H NMR spectrum of ergosterol peroxide (11) in $CDCl_3$

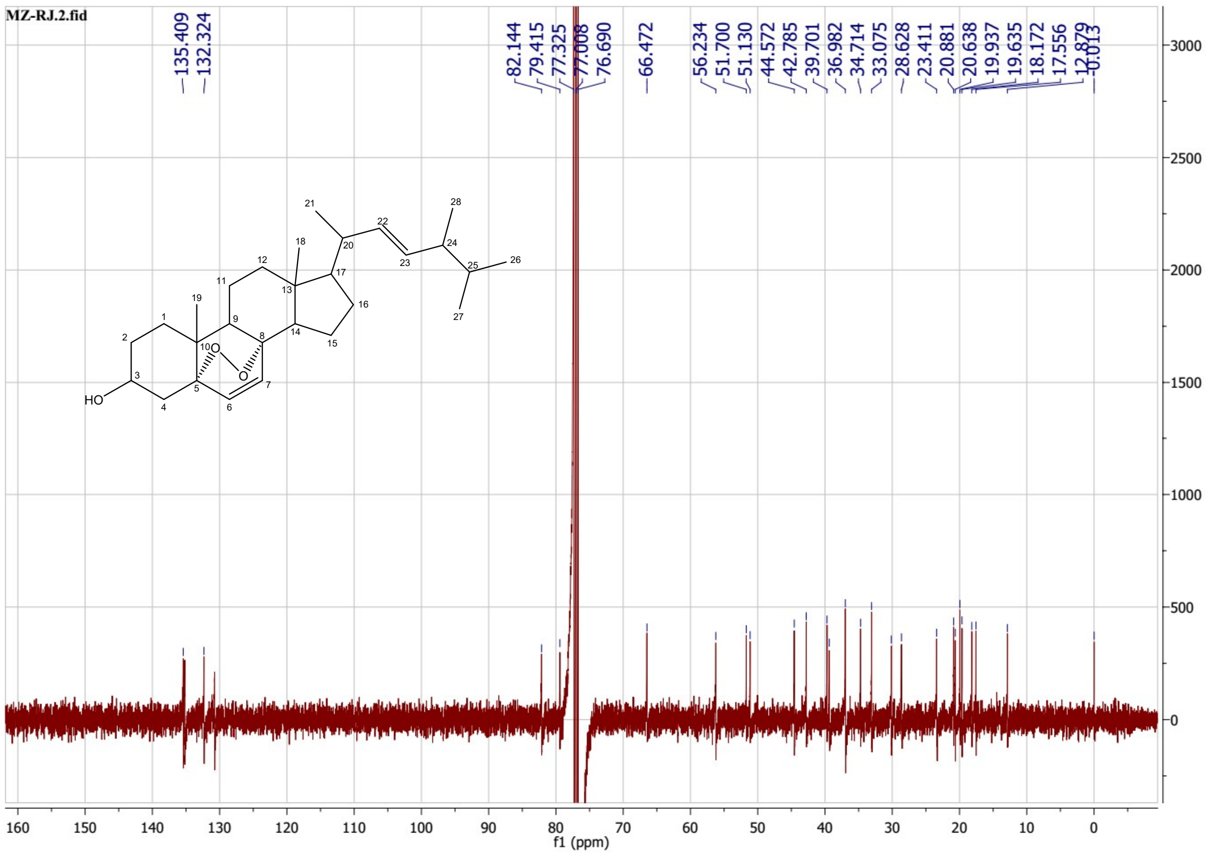

Fig. S35. ^{13}C NMR spectrum of ergosterol peroxide (11) in $CDCl_3$

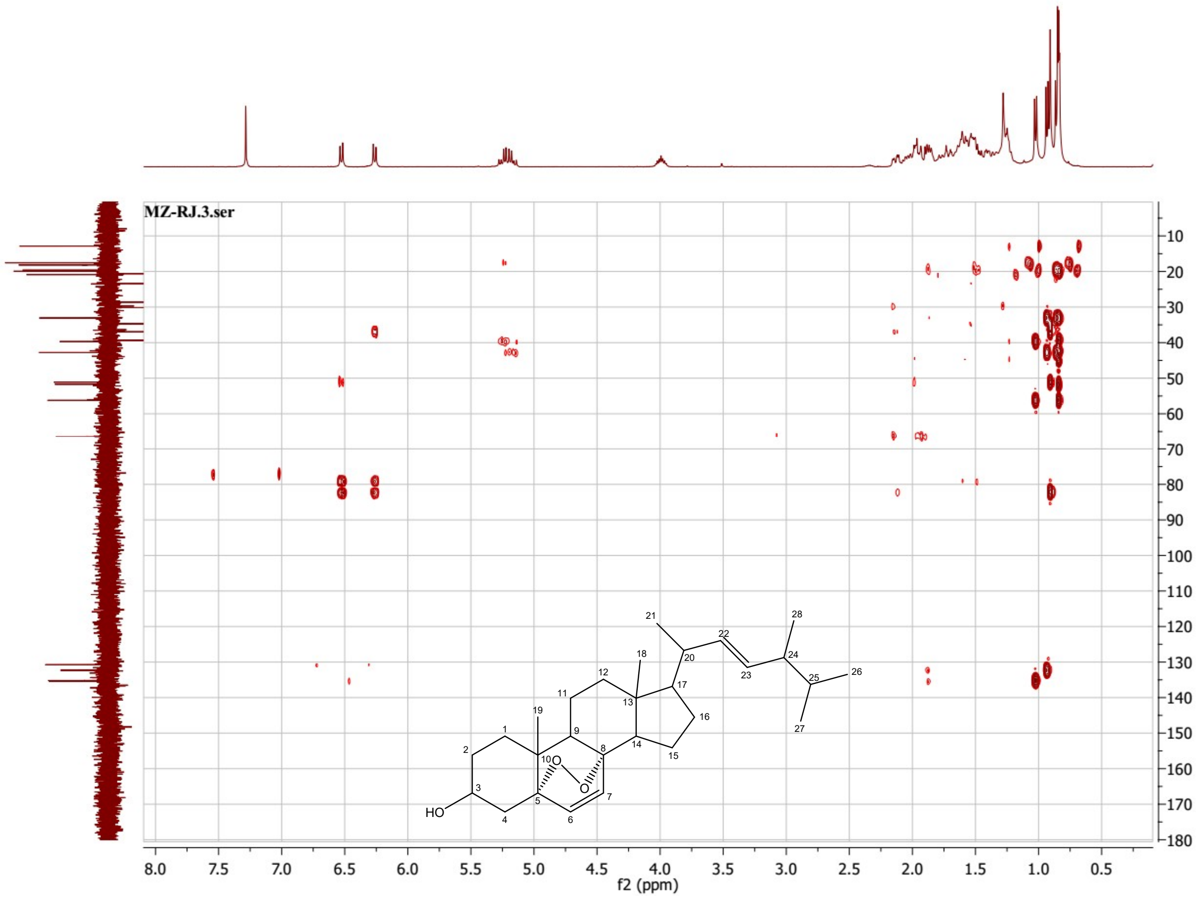

Fig. S36. HSQC spectrum of ergosterol peroxide (11) in $CDCl_3$

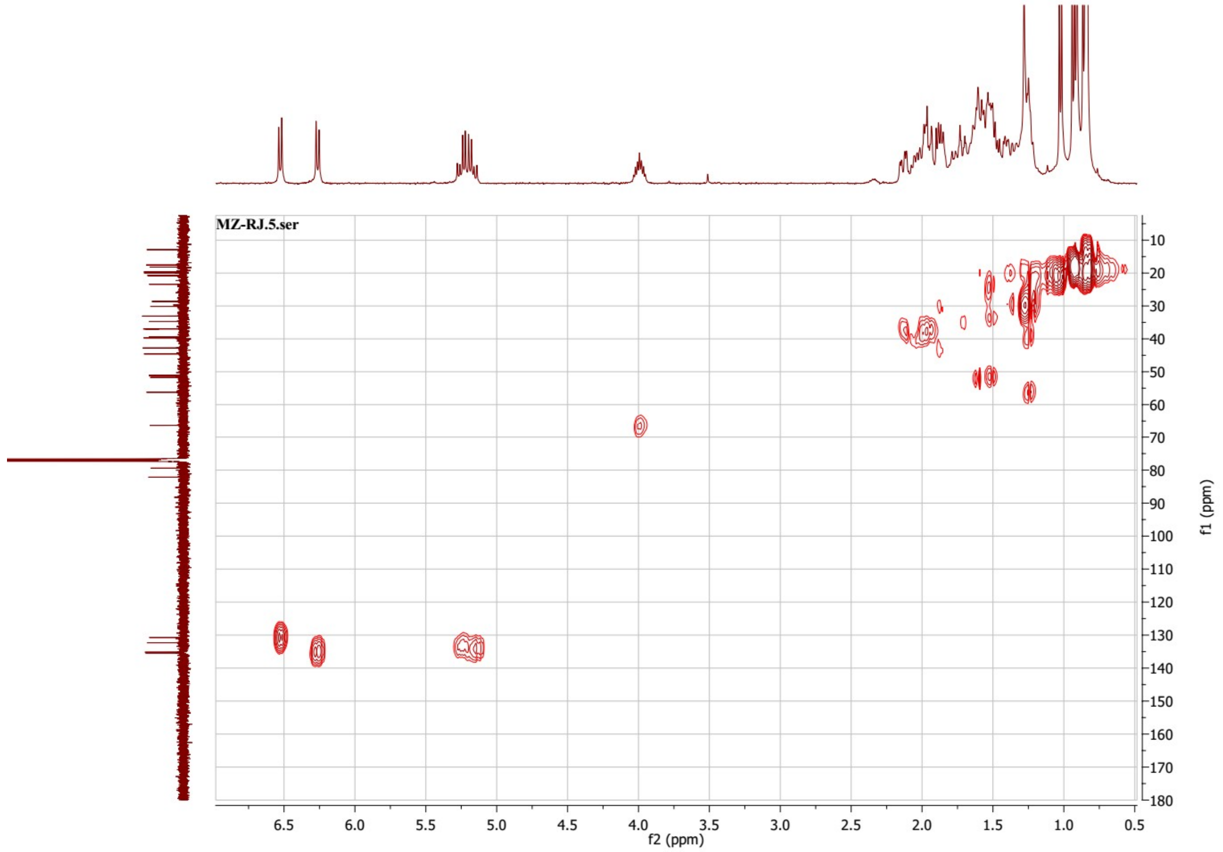

Fig. S37. HMBC spectrum of ergosterol peroxide (11) in $CDCl_3$

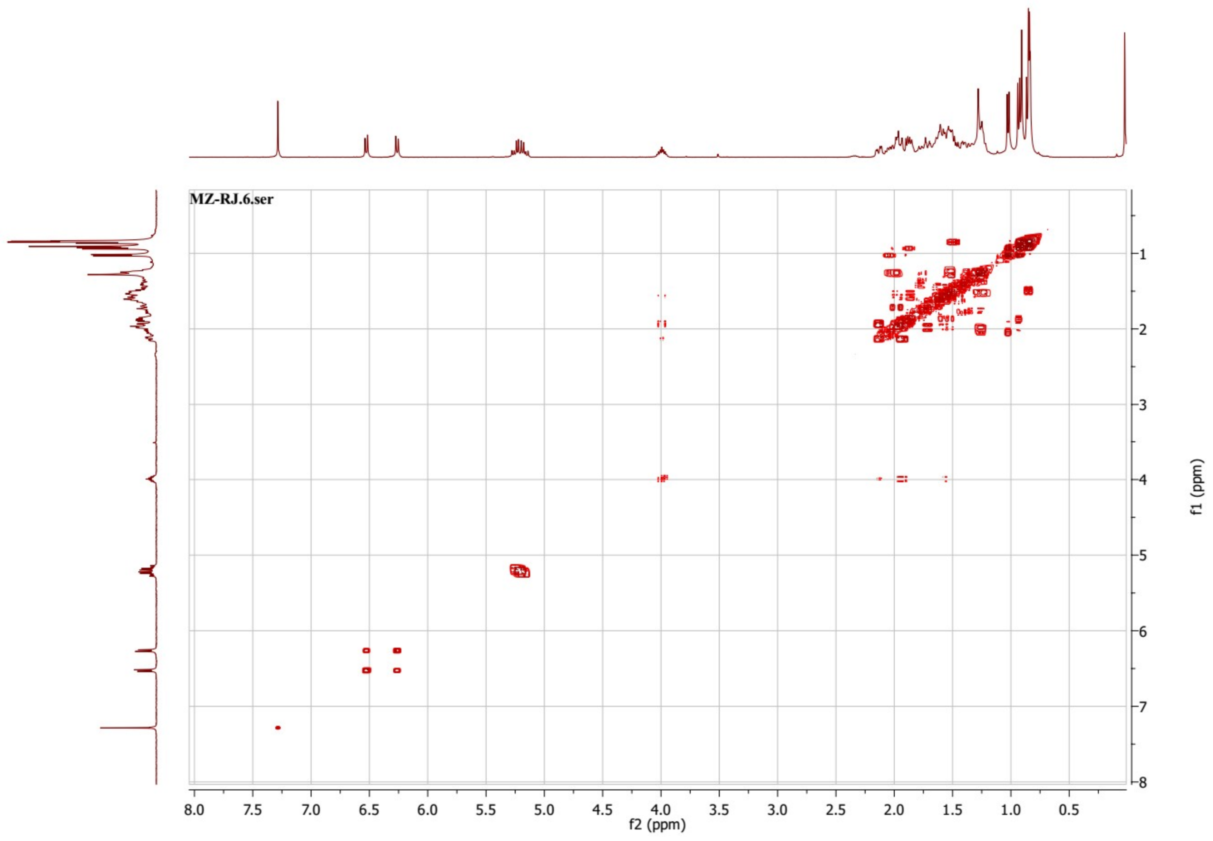

Fig. S38. COSY spectrum of ergosterol peroxide (11) in $CDCl_3$

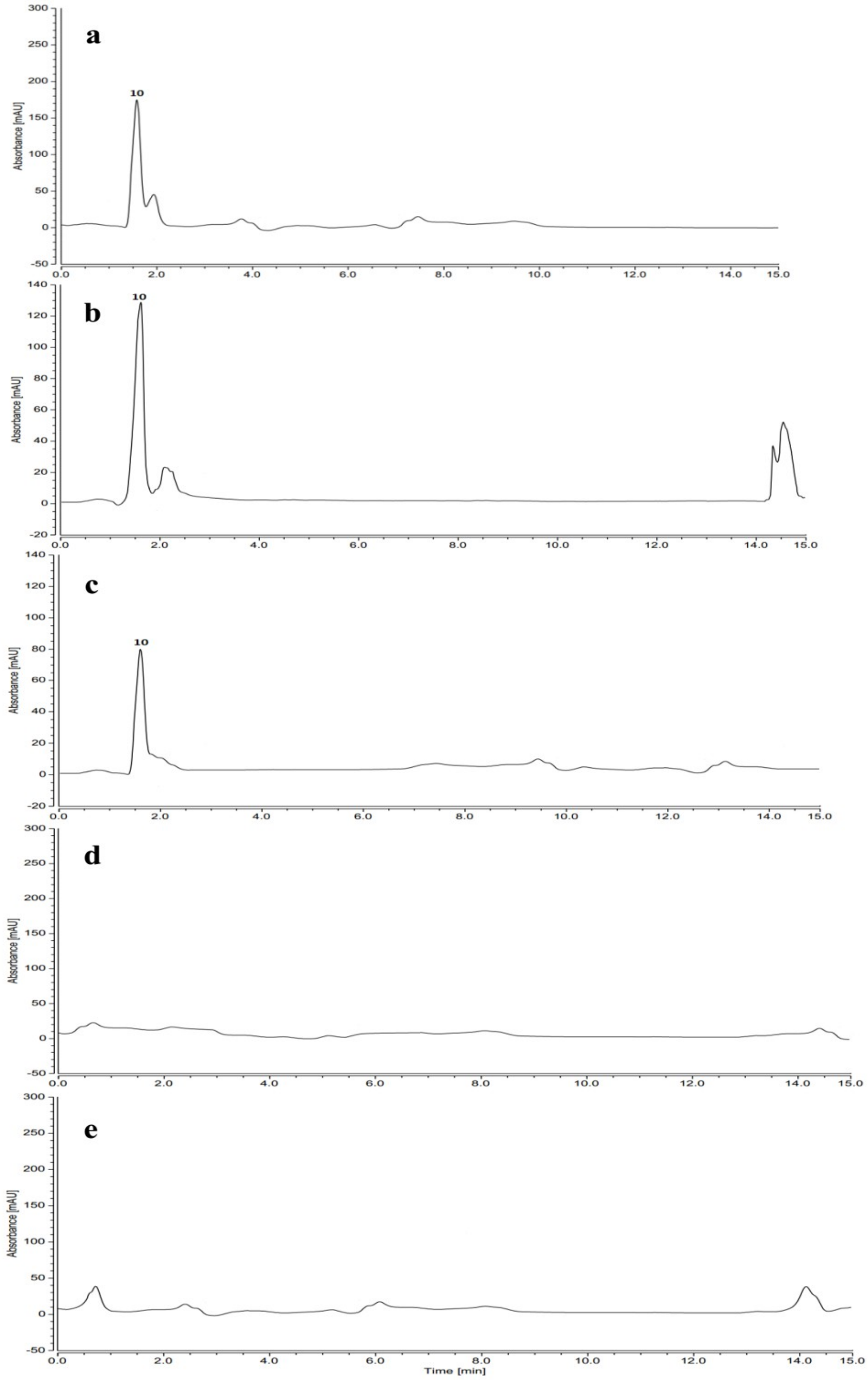

Figure S39. HPLC profiles (detected at 254 nm) of fungal extracts obtained from different culture media. (a) rice; (b) sabouraud Dextrose; (c) Czapek Dox; (d) malt extract; (e) malt extract + salt.

Table S1. Results of the ten top-scored 3D hypothetical pharmacophores generation with information of statistical significance.

Hypothesis	Score	F value	degree of freedom
Hypo 1	72.82	-108.99	0.0
Hypo 2	71.54	-101.46	7.52
Hypo 3	69.74	-101.70	7.28
Hypo4	65.56	95.43	13.56
Hypo 5	64.83	95.20	13.78
Hypo 6	63.27	92.67	16.31
